# Genome-wide data reveal cryptic diversity and genetic introgression in an Oriental cynopterine fruit bat radiation

**DOI:** 10.1186/s12862-016-0599-y

**Published:** 2016-02-18

**Authors:** Balaji Chattopadhyay, Kritika M. Garg, A. K. Vinoth Kumar, D. Paramanantha Swami Doss, Frank E. Rheindt, Sripathi Kandula, Uma Ramakrishnan

**Affiliations:** School of Biological Sciences, Madurai Kamaraj University, Madurai, India; Ecology and Evolution, National Centre for Biological Science, TIFR, Bangalore, India; Department of Biological Sciences, National University of Singapore, Singapore, Republic of Singapore

**Keywords:** Gene flow, SNPs, ddRAD, *Cynopterus sphinx*, *Cynopterus brachyotis*

## Abstract

**Background:**

The Oriental fruit bat genus *Cynopterus*, with several geographically overlapping species, presents an interesting case study to evaluate the evolutionary significance of coexistence versus isolation. We examined the morphological and genetic variability of congeneric fruit bats *Cynopterus sphinx* and *C. brachyotis* using 405 samples from two natural contact zones and 17 allopatric locations in the Indian subcontinent; and investigated the population differentiation patterns, evolutionary history, and the possibility of cryptic diversity in this species pair.

**Results:**

Analysis of microsatellites, cytochrome *b* gene sequences, and restriction digestion based genome-wide data revealed that *C. sphinx* and *C. brachyotis* do not hybridize in contact zones. However, cytochrome *b* gene sequences and genome-wide SNP data helped uncover a cryptic, hitherto unrecognized cynopterine lineage in northeastern India coexisting with *C. sphinx*. Further analyses of shared variation of SNPs using Patterson’s D statistics suggest introgression between this lineage and *C. sphinx*. Multivariate analyses of morphology using genetically classified grouping confirmed substantial morphological overlap between *C. sphinx* and *C. brachyotis*, specifically in the high elevation contact zones in southern India.

**Conclusion:**

Our results uncover novel diversity and detect a pattern of genetic introgression in a cryptic radiation of bats, demonstrating the complicated nature of lineage diversification in this poorly understood taxonomic group. Our results highlight the importance of genome-wide data to study evolutionary processes of morphologically similar species pairs. Our approach represents a significant step forward in evolutionary research on young radiations of non-model species that may retain the ability of interspecific gene flow.

**Electronic supplementary material:**

The online version of this article (doi:10.1186/s12862-016-0599-y) contains supplementary material, which is available to authorized users.

## Background

Understanding and identifying cryptic diversity directly informs conservation and plays a crucial role in the formulation of management decisions [1, 2]. This is particularly important for species pairs that show geographical as well as morphological overlap [3–6], wherein taxonomic identification remains a difficulty and evolutionary processes go undetected [5–7]. In such cases a composite approach of supplementing morphological identification with genetic classification considerably improves species identification, detects cryptic diversity, thereby providing a better inventory of natural history and biodiversity [2, 5–9]. Such an approach also acts as a primer to understand evolutionary trajectories of codistributed species pairs and behavioral as well as ecological contingencies of coexistence.

Restriction digestion based genome-wide data often retrieve thousands to hundreds of thousands of loci and in recent times have provided unparalleled resolution towards the understanding of genetic diversity, gene flow and evolutionary history, specifically of non model organisms. For example, using a few million base pairs of sequence data, Wagner et al. [10] were able to differentiate between various lineages of Lake Victoria cichlids, which diversified only 15,000 years ago. Other genomic scans have revealed the importance of transposable elements in maintaining different butterfly races [11], parallel phenotypic evolution in sticklebacks involving similar regions of the genome [12], rare introgression [13] and inbreeding [14]. Genome-wide data also shows great promise towards the discovery and understanding of cryptic diversity, and evolutionary studies of non-model organisms [2, 9, 15].

Old world fruit bats of the genus *Cynopterus* present an interesting natural system to study evolutionary dynamics of codistributed species pairs. The genus *Cynopterus* has undergone a recent, relatively rapid radiation giving rise to species complexes whose phylogeny remains unresolved [3, 16]. Many of these lineages share broad zones of coexistence across south and southeast Asia [3]. Most cynopterine species show considerable overlap in niche space and morphology, as diets are simple and non-specialized [17]. Species level identification of cynopterine fruit bats remains a problem, especially in contact zones [17–20] and genetic diagnosis remains a necessity, especially in the absence of extensive collections and detailed morphological information from many regions.

In the present study, we assessed morphological and genetic diversity of two congeneric cynopterine fruit bats, *Cynopterus sphinx* and *C. brachyotis*. These species are closely related [3] and show broad morphological overlap in areas of coexistence [3, 19, 20]. We characterized zones of overlap between these two species using morphological, population genetic and phylogenetic analyses. We examined morphological variation based on species-specific phenotypic characters (following [18, 20]), obtained genetic classification of our dataset without a priori assumption of group membership using autosomal microsatellite markers and up to ~10,000 SNPs derived from genome-wide data (double digest restriction site associated DNA sequencing, ddRADseq following [21]) and applied mitochondrial cytochrome *b* (cyt*b*) gene sequences along with upto ~700,000 bp of sequence data derived from ddRADseq to reconstruct the species phylogeny. Using our genome-wide dataset, we also tested for the effect of missing data and an increase in the number of loci in genetic assignment and phylogeny reconstruction. We further used classifications based on genetic markers to assess the extent of morphological overlap between both species and generated classification functions for morphological variables that can be used for field identification of cynopterine bats in India.

We document the presence of a cryptic cynopterine lineage and reveal introgression between deeply diverged species-level cynopterine lineages in northeastern India. We also demonstrate that genome-wide data spanning thousands of loci are robust to the effects of missing data. Lastly, our phenotypic examinations have failed to come up with diagnostic morphological traits for species level classification in the contact zones and we suggest that genetic data, specifically genome-wide SNPs should be used for species identification.

## Methods

### Ethics statement

This study and sampling protocols were approved by and were in accordance with the institutional ethics committees (Internal Research Review Board (IRB), Ethical Clearance (EC), Biosafety and Animal Welfare committee approval to Balaji Chattopadhyay dated 21-11-2005 Madurai Kamaraj University and Institutional Animal Ethics Committee (IACE) to Uma Ramakrishnan (UR-3/2009), National Centre for Biological Sciences). The study species are not endangered and are classified under Least Concern category in the International Union for Conservation of Nature (IUCN) red list.

### Sampling

**Fig. 1:**
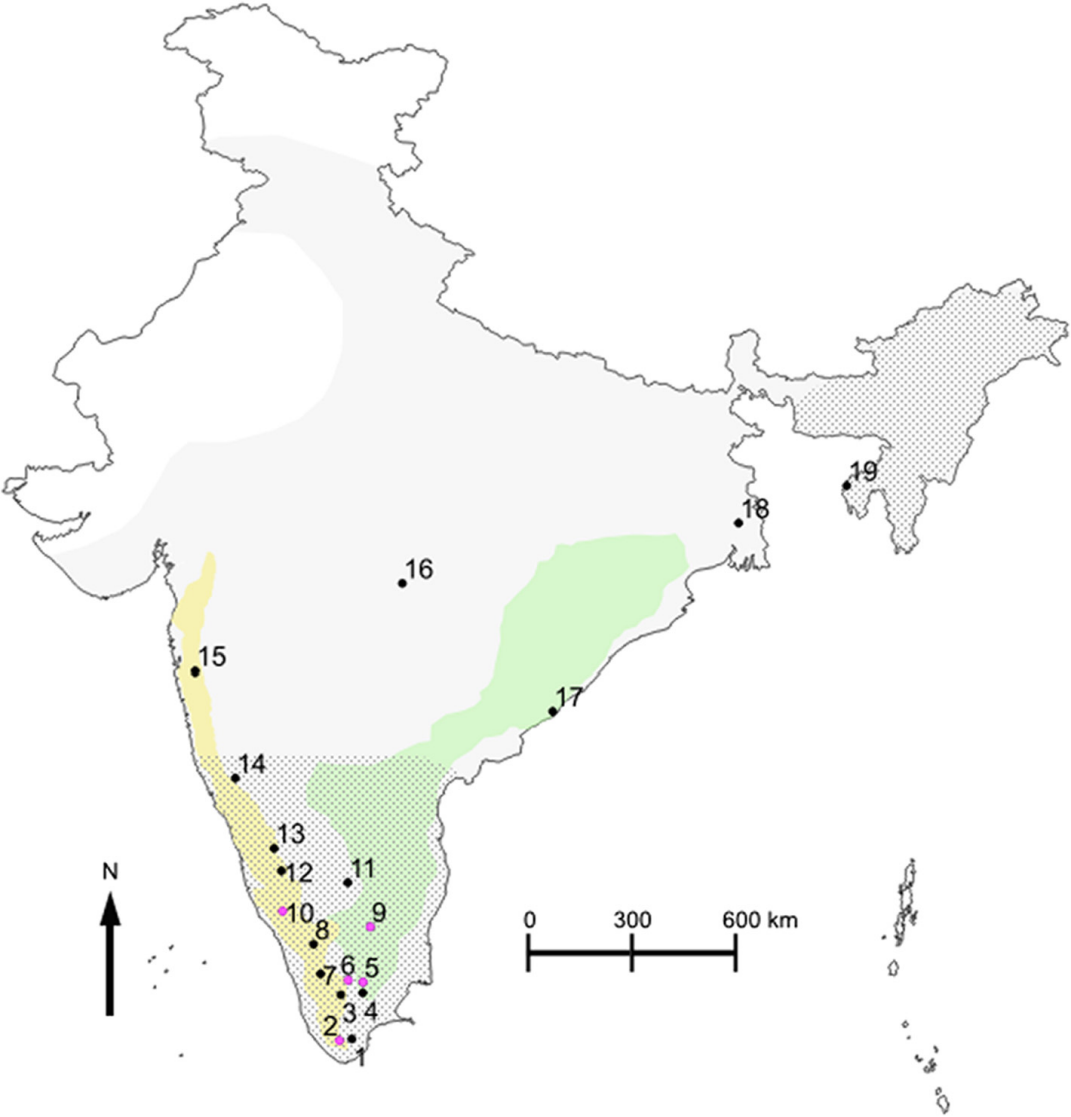
Map showing the distribution and sampling details of each species. Shaded area corresponds to the range of *C. sphinx* across India. Dotted areas correspond to the range of *C. brachyotis* lineage across India. Distribution maps for both species were obtained from International Union for Conservation of Nature. Rajasri Ray provided the shape files of the political boundary of India as well as the boundaries of the Eastern Ghats and Western Ghats. While *C. sphinx* is found across various habitat types at lower altitudes, *C. brachyotis* is restricted to mountain ranges [18]. In southern India, *C. brachyotis* is reported from the Eastern Ghats and the Western Ghats mountain ranges ([18] and the present study). In the map, we have denoted the Eastern Ghats in green and the Western Ghats in yellow. Sampling locations are presented as black dots with numbers corresponding to a population. Locations corresponding to areas of coexistence are marked in pink dots. Details of sampling are provided in Additional file 1: Table S1

We sampled bats between August 2005 and February 2012 at 19 locations across India (Fig. 1 and Additional file 1: Table S1). The study species are considered as vermin under the Indian Wildlife protection Act (1972). These are not protected species and sampling them outside reserve forest limits does not require any permission. We performed sampling following forest department permit (WL5/44926/2010, dated: 11.03.2011) within reserve forest limits (locations: 2 and 7, Additional file 1: Table S1). All other sampling locations were outside reserve forest limits and we obtained oral permissions from owners whenever we sampled in private areas.

We captured bats either in hoop nets at their day roost or in mist nets (Avinet Inc., USA) at foraging grounds after nightfall. We classified individuals as juveniles or adults based on the extent of tooth wear of the upper canines, pelage colouration and ossification of epiphyseal bones [20, 22–24]. We used only adult individuals for morphometric analyses, whereas all individuals were subjected to molecular analyses.

We measured lengths of the forearm, tibia and ear of each bat using a dial caliper (Avinet Inc, USA). We also used ear margins as a categorical variable by coding it as distinct, faint or absent. We initially identified bats in the field as *C. sphinx* or *C. brachyotis* following Bates and Harrison [18] and Storz and Kunz [20]. However, we also frequently observed bats with a long forearm length (characteristic of *C. sphinx*), but with either pale or no ear margin (characteristic of *C. brachyotis*). Because of this general lack of consensus regarding morphological characters we used the following scheme for field identification based on our prior field experience in the southern Western Ghats. We gave major weightage to the presence of an ear margin, assigning to *C. sphinx* all bats with a prominent ear margin and to *C. brachyotis* those with no margin. Similarly, we assigned to *C. sphinx* all bats with a forearm length ≥63.4 mm. Individuals that could not be identified as either species were labeled ‘unassigned’. We described an area as a contact zone or zone of coexistence when morphologically typical adult individuals of both species were captured either in the same mist net or during multiple sampling sessions in the same location. We used published records [18] alongside our sampling observations to identify locations as allopatric.

We obtained tissue biopsies using a 6 mm or 4 mm sterile punch from both wing patagia of an individual and stored them in 95 % ethanol (- 20 °C) prior to DNA extraction. All bats captured in mist nets were released immediately after sampling. Bats captured in day roosts were treated following Garg et al. [23]. All sampling protocols were in accordance with the ethical standards of the institutions involved in this research.

### DNA extraction and microsatellite genotyping

We extracted total genomic DNA using a modified salt-chloroform extraction protocol following Chattopadhyay et al. [22]. We amplified three tri- and six tetra- nucleotide repeat loci, previously developed for *C. sphinx* [25], either using Ampli-Taq Gold DNA polymerase (Applied Biosystems, n = 266) following Chattopadhyay et al. [22] or PCR Master mix (MM, Qiagen, n = 121) (Additional file 2). We genotyped all samples using the ABI3100 XL platform and scored allele sizes using Genemapper v 4.0 (Applied Biosystems). We normalized post genotyping allele sizes using TANDEM [26], which uses a power function to transform allele sizes to integers, while minimizing the rounding errors. Details of error rates and missing data calculations are provided in the supplementary information. Percentage of missing data, number of alleles and allele size range are summarized in Additional file 3: Table S2. We used the normalized allele sizes for subsequent analyses.

### SNP generation from ddRAD libraries

In addition to genotyping microsatellites, we also mined SNPs from genome-wide data using the ddRAD approach [21] (details in Additional file 2). We chose a subset of samples from both species as well as putative introgressed individuals following trends obtained from the microsatellite data (Additional file 1: Table S1). We digested these samples with NlaIII and MluCI enzymes. We used 130 ng to 200 ng of DNA as starting material. Details of library preparation are provided in the Additional file 2. A paired end run in one lane of an Illumina Hiseq1000 was performed with 10pM product from each library. Quality scores (QC) (FastQC, [27]) suggested poor quality in the restriction sites due to low diversity in both the forward and paired end run (Additional file 4: Figure S1). We analyzed only the forward run as we wanted to mine unlinked markers and obtain only a single SNP per locus. We used the STACKS 1.09 [28] pipeline for demultiplexing as well as to obtain SNPs (process_radtag .pl, denovomap.pl and populations.pl). We allowed for one error in the barcode during demultiplexing and trimmed the dataset to 80 bp length. We used the denovomap.pl program in STACKS to call SNPs. The minimum number of reads to call a stack (stack depth) was set at 10 (-m). Unusually high numbers of identical reads signify repeat regions or regions of gene duplications. In order to remove these regions we allowed breakup of highly repetitive stacks (-t). We allowed for 2 mismatches between the reads within a stack (-M) of an individual and further allowed two mismatches between stacks when comparing across individuals. SNP calling was based on default parameters. We used the population.pl script to generate unlinked SNPs with varying degrees of missing data across individuals. We included a SNP when the locus containing it was present in at least one species, ensuring that both species contribute towards marker generation. This would reduce the effect of ascertainment bias arising from restriction site polymorphism (enhancing intraspecific variation), and account for reduced variability (SNPs are isolated *de novo* from both species simultaneously).

### Mitochondrial sequencing

We sequenced the entire (1140 bp) cyt*b* gene from a subset of pure individuals of both species as well as unassigned individuals (112 sequences; KF042154-KF042252, KJ417498-KJ417512). We used a suite of generic primers as well as specific primers designed for this study, to amplify the entire gene (Additional file 2).

### Morphological analysis

We obtained summary statistics of morphological variables. We investigated the morphological variation within our dataset through multivariate analyses of morphological traits. We tested for multivariate normality in R [29]. We performed multivariate analyses of the morphological variables in the R package FactomineR 1.27 [30]. We performed PCA using the continuous variables (forearm length, tibia length and ear length). However, we also incorporated the categorical variable ear margin as a supplementary variable to improve clustering [30]. We only considered individuals without missing data for this analysis.

### Microsatellite analysis

We obtained allele numbers and allele size ranges, performed a test for deviation from Hardy Weinberg equilibrium (HWE) (Genepop 4.2.1, [31]) and tested for Linkage Disequilibrium in FSTAT 2.9.3.2 [32] (Additional file 2). We checked for the presence of null alleles in the dataset using Microchecker [33], and tested for the presence of homoplasy and ascertainment bias within our dataset (Additional file 2). We performed a test for neutrality in BayeScan 2.1 [34] with default settings. The algorithm divides F_ST_ into a population specific (beta) and a locus specific component (alpha). It looks for significant deviation of the locus specific component from the population specific component. A significant alpha value would suggest that the locus is under selection. We set a prior odd of 10 assuming that the neutral model is 10 times more likely than the selection model at a locus. We used a 5 % cutoff value for the false discovery rate to identify outlier loci. We performed all analyses using genetically pure individuals obtained from an initial assignment test as mentioned below.

We used a model-based clustering approach implemented in STRUCTURE 2.3.4 [35] to identify genetic clusters (K) within our dataset and to quantify the extent of admixture based on our microsatellite loci. STRUCTURE runs were implemented without an a priori assumption of group membership. We ran STRUCTURE from K 1 to 6 with 10 iterations per K. For each iteration we implemented a burnin of 500,000 generations and MCMC for 1,000,000 generations. We first used the second order rate of change of the log probabilities of the data (delta K, [36]) to statistically identify the most likely number of genotypic clusters (K) within the entire dataset. Further, for each K that we obtained, we evaluated individual ancestry coefficients (q values) to assign individuals into population clusters using CLUMMP 1.1.2 [37]. We performed a full search for K = 2 and 3, and used the greedy algorithm (10,000 iterations) for higher K values.

To assess the capability of our microsatellite loci to distinguish between species and to obtain cutoff values for ancestry coefficients (q values) for pure and admixed individuals, we generated a simulated dataset in Hybridlab [38] and followed Burgarella et al. [39] to obtain estimates of efficiency, accuracy, and type I errors in assigning purebreds and hybrids using STRUCTURE (Additional file 2).

### Genome-wide SNP analysis

We calculated average heterozygosity in Cervus 3.0 [40] and extent of missing data in PLINK [41]. We also tested for deviation from neutrality in BayeScan 2.1 as mentioned earlier and subsequently performed individual assignments in STRUCTURE using only neutral loci (using same conditions as microsatellite analysis). We ran STRUCTURE from K = 1 to 6 with 10 iterations per K. Every iteration included a burnin of 50,000 generations and MCMC for 100,000 generations. We obtained the optimal K using Evanno’s method [36] similar to the microsatellite data.

To test the effect of missing data and number of loci, we obtained SNPs from STACKS with different levels of missing data and assessed trends observed for each dataset. We mined loci if they were present at least in one species; thus the extent of missing data allowed per locus reflects the species level missing data and not the level of missing data in the entire dataset. In this way, we obtained four datasets with the following levels of missing data allowed: 10 %, 30 %, 50 % and 70 %.

### Mitochondrial DNA-based phylogenetic reconstruction

We aligned our 112 full length cyt*b* sequences with previously published *Cynopterus* sequences (n = 12; *C. sphinx*: FJ489964, FJ489958, JX283292, DQ445703, FJ489972; *C. brachyotis* Sunda lineage: GU724956; *C. brachyotis* Phillipines lineage: AB046320, AB046321; *C. brachyotis* Forest lineage: GQ410210 and *C. horsfieldii*: EF201637, EF201639 and EF201643) and outgroup taxa (n = 7; *Ptenochirus* jagori: FJ218480 and GQ410211; *Pteropus vampyrus*: EF584230 and JN398212; *Rhinolophus ferrumequinum*: EU436673; *Hipposideros bicolor*: DQ054808 and *Megaderma lyra*: DQ888678; alignment length of 996 bp, Additional file 2) and reconstructed the phylogeny of the *Cynopterus* species complex using a Bayesian paradigm implemented in MrBayes 3.2.5 [42]. We performed two runs with four chains. The swapping frequency and temperature were kept at default values. Trees were sampled every 500^th^ generation and diagnostics were calculated every 5,000^th^ generation for a total of 10,000,000 generations per run. At this point the standard deviation of the split frequency had reached below 0.01. We used Tracer v1.5 [43] to check for convergence. We obtained consensus trees from MrBayes using a 50 % burnin and the resultant phylogram was viewed in Figtree 1.4 [44]. Some of the published sequences were very short (690 bp), specifically the Myanmar lineage of *C. brachyotis* (AY628945), which is represented by only one sequence [3]. To accommodate these sequences and obtain a wider coverage of cynopterine lineages we added these sequences to our dataset and reconstructed a second phylogeny (based on a 690 bp alignment; other GenBank accession id’s included: *C. sphinx* Myanmar lineage: AY629000; *C. brachyotis* Sunda lineage: AY628945; *C. brachyotis* Sulawesi lineage: AY628937 and AY628938; *C. brachyotis* Forest lineage: AY628966) in MrBayes using the same conditions. This second analysis was run for 5,000,000 generations.

We computed mean between-group and within-group genetic distance in MEGA 5.0 [45]. We also tested for homoplasy (saturation of phylogenetic information) within our dataset using DAMBE [46] (Additional file 2). We did not observe any significant saturation within our dataset (Additional file 3: Table S3).

### Genome-wide locus-based phylogenetic analysis

We also performed a phylogenomic reconstruction with the genome-wide ddRAD dataset. We first isolated concatenated sequence data from the 46 individuals using the pipeline pyRAD [47]. We used demultiplexed raw reads obtained from STACKS (process_radtags.pl) as an input to pyRAD. Thus, data obtained from this pipeline was independent from the data obtained from the STACKS pipeline. We considered the minimum depth at each locus for an individual as 10, restricted the number of undetermined bases allowed per locus to 4 and set the similarity threshold for global and within sample clustering at 0.88.

We generated four distinct datasets allowing for 10 %, 30 %, 50 % and 70 % missing data (sequence length for each dataset: 770 bp; 9,064 bp; 110,642 bp; and 700,088 bp, respectively). Unlike for the SNP dataset, the missing data cutoff here reflects the actual missing data allowed per locus across all 46 individuals.

We followed a supermatrix approach with concatenated sequence data to reconstruct the phylogeny of our sampled species using maximum likelihood as implemented in RAxML v8 [48]. We used the GTR + gamma model of sequence evolution and performed a single full maximum likelihood tree search, applying the rapid bootstrap algorithm with 1000 replicates to each dataset. The final unrooted tree was viewed in Figtree with midpoint rooting.

### Test for introgression

We obtained Patterson’s D statistic to test if a pattern of shared variation between groups can be better explained by gene flow than incomplete lineage sorting [49]. This test has been specifically useful in identifying incidents of introgression using SNP based genome-wide datasets [13]. We performed the four taxa D test in the evobiR package in R. This test assumes that the data consist of four clades: two sister clades, one putative admixed clade formed due to possible gene flow between the sister clades and an outgroup clade. It then assesses the shared variation across all SNPs (which are homozygous in the outgroup) that follow an ABBA or BABA pattern between these clades [13, 49–51]. We performed 1000 bootstraps to calculate the standard deviation of the D-statistic and calculated Z-scores to determine significant introgression (Z-score > 2.55 and p value of two tailed test < 0.01). We performed the test for introgression for all four levels of missing data generated in pyRAD.

### Discriminant analysis and classification function

In order to understand if genetic classifications can improve morphological identification of both species, we performed a forward stepwise discriminant analysis (DA, STATISTICA version 10) using genetic data as the dependent variable and the three continuous variables (forearm length, ear length and tibia length) as independent predictors. We performed classification of cases and obtained classification functions.

## Results

### Field sampling and morphological analysis

We captured and sampled 405 bats (395 adults: *C. sphinx* - 230, *C. brachyotis* - 138 and unassigned - 27) across their ranges in India (Fig. 1, Additional file 1: Table S1). Initial field identifications revealed the presence of both morphologically typical as well as unassigned individuals (Additional file 1: Table S1). We identified two distinct contact zones, one in the Eastern Ghats mountain range (Yercaud, location: 9, Fig. 1) and the other in the southern Western Ghats mountain range (KMTR, Kalakad Mundanthurai Tiger Reserve, location: 2, Fig. 1). In these areas, we often captured both species in the same mist net suggesting an overlap in foraging habitats. We removed juveniles from all morphological analyses (Additional file 1: Table S1).

**Fig. 2:**
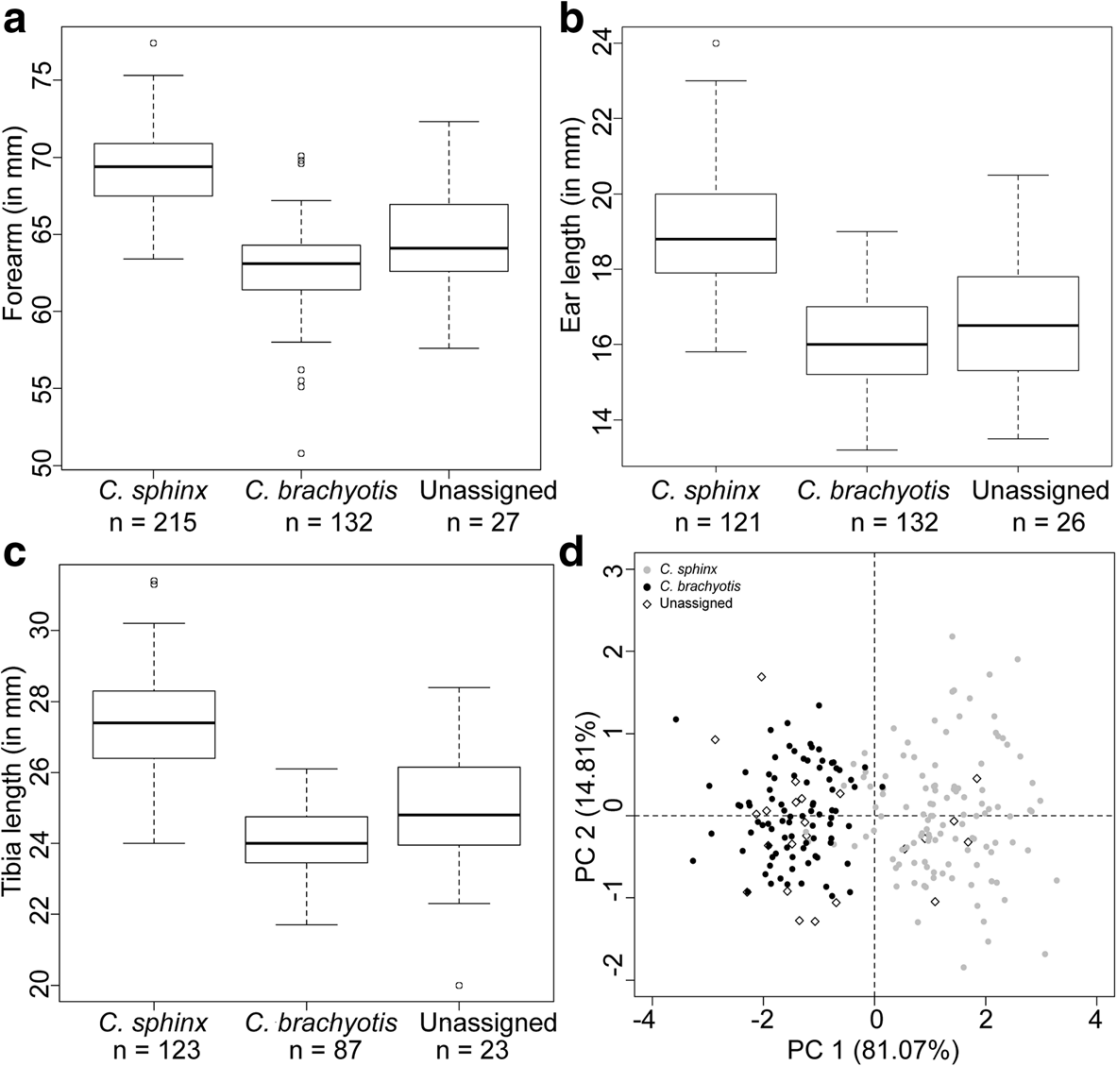
Boxplots representing morphological variation of the following traits: **a**) forearm length, **b**) ear length and **c**) tibia length. **d**) Multivariate analysis of all morphological variables

Morphologically ‘unassigned’ individuals were mostly captured throughout the higher elevation distributional range of *C. brachyotis* (average elevation = 1137.6 m, Additional file 1: Table S1). We observed considerable overlap in morphological variables between the two species (Fig. 2 a, b and c, Additional file 3: Table S4). We did not observe sexual dimorphism in these species (Additional file 3: Table S5). The morphological variables were not normally distributed (Jarque-Bera multivariate normality test, *P* < 0.001). PCA analysis based on data from 228 individuals for which we had no missing data resulted in the first two axes (which explained 96 % of variation; Fig. 2d) differentiating samples into two major clusters with overlap: *C. sphinx* and *C. brachyotis* (Fig. 2d). Among the continuous variables, forearm length was the best predictor in the first axis and was strongly correlated with tibia length, followed by ear length (Additional file 5: Figure S2). Ear length contributed most in the second axis (Additional file 3: Table S6).

### Microsatellite analysis

We genotyped 387 individuals, and overall, our dataset had 0.4 % missing data (Additional file 3: Table S2) affecting eight *C. sphinx* and four *C. brachyotis* individuals. Twelve populations out of 19 showed a significant heterozygote deficit, though there was no significant linkage disequilibrium within our dataset. We observed null alleles at various loci within our samples (Additional file 6: Table S7), especially in CSP8 (10 out of 19 populations). We observed homoplasy in our microsatellite dataset, but no ascertainment bias (Additional file 2). We also observed that four loci (CSP2, CSP7, CSP8 and CSP9, Additional file 3: Table S8) did not fit neutral expectations. We therefore performed microsatellite-based analyses both with and without these loci.

**Fig. 3:**
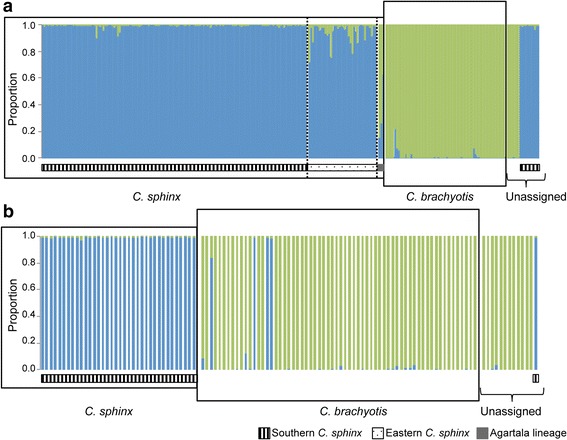
Barplots of the ancestry coefficient q at K = 2 (all 9 loci) of individuals belonging to **a**) populations in allopatry and **b**) populations in contact zones using microsatellite markers (assignment test with all 9 loci). The vertical axis represents the proportion of the ancestry coefficient of each individual belonging to each species. Intraspecific subdivisions within *C. sphinx* and the Agartala lineage that were identified from the analyses of SNP data (see text for details) are highlighted

STRUCTURE runs with all nine loci revealed that both species neatly segregate into two clusters and that, statistically, K = 2 best explains the data (Fig. 3a, b and Additional file 7: Figure S3). One cluster included most of the field identified *C. sphinx* and the other *C. brachyotis*. We further evaluated the average ancestry coefficients (q) of K = 2. We used cutoff values for pure individuals of > 0.85 and < 0.15 and for intermediates of ≤ 0.85 and ≥ 0.15 based on the values obtained from the simulated data (Additional file 2). We observed genetically admixed individuals mainly within allopatric populations (n = 7) of both species (Additional file 3: Table S9). Conversely, we observed only one admixed individual in a contact zone (Fig. 3b). None of the genetically admixed individuals had any missing data. We observed that all field based unassigned individuals could be genetically assigned to either of the two species (Fig. 3a, Additional file 3: Table S10A).

STRUCTURE runs using only neutral loci (cutoff values for pure individuals: > 0.70 and < 0.30, and for intermediates: ≤ 0.70 and ≥ 0.30, Additional file 2) also revealed the presence of 8 genetically admixed individuals. However, there was broad disagreement in assigning admixed individuals between both datasets. Analyses with the five neutral loci revealed additional admixed individuals in the Tirunelveli population which were considered pure in analysis with all loci. Conversely, some individuals which were considered admixed with all loci emerged as pure when including only neutral loci (Additional file 3: Table S9).

Microsatellite analysis also revealed the presence of additional contact zones in the hill ranges of the Western and Eastern Ghats (locations: 5, 6 and 10, Fig. 1 and Additional file 3: Table S10A). Genetic assignment test revealed the presence of *C. sphinx* in high altitude regions of both the Western Ghats and the Eastern Ghats (average elevation = 1168.67 m). Significantly, we observed that almost the entire sampled distributional range of *C. brachyotis* is part of the contact zone.

Comparisons across various K values revealed two admixed genomic clusters within *C. sphinx* at K = 3 and K = 4, one cluster representing samples from Eastern India (eastern *C. sphinx*, locations: 17, 18 and 19 in Fig. 1 and Additional file 1: Table S1) and the other cluster representing samples from peninsular and southern India (southern *C. sphinx*, locations: 1, 2, 4, 5, 6, 9, 10-16 in Fig. 1, Additional file 1: Table S1) (Additional file 8: Figure S4A). Further increase in K did not reveal any additional biologically clusters (data not shown). When the STRUCTURE analysis was repeated with only neutral loci, we did not observe any biologically relevant sub-structuring (Additional file 8: Figure S4B) possibly suggesting the lack of power of these five loci to identify intraspecific variation.

### Genome-wide SNPs

We obtained 274 million paired-end reads from 46 individuals. In our selection for the ddRADseq data subset, we chose individuals representative of the microsatellite diversity within all species-level lineages based on results from microsatellite STURCTURE analysis. We analyzed only the forward reads (140 million reads), out of which 84 million reads passed the quality filtering criteria (process_radtags). The number of reads per individual ranged from 0.8 million reads to 3.1 million reads, with an average of 1.8 million reads. We obtained 70, 365, 2381, and 10,866 SNPs allowing for 10 %, 30 %, 50 % and 70 % missing data, respectively. We detected signs of selection in three loci from the 30 % missing data dataset and in 20 loci from the 50 % missing data dataset, but none in the other two datasets. The average heterozygosities and levels of missing data per locus and per individual are summarized in the Additional file 3: Table S11.

**Fig. 4:**
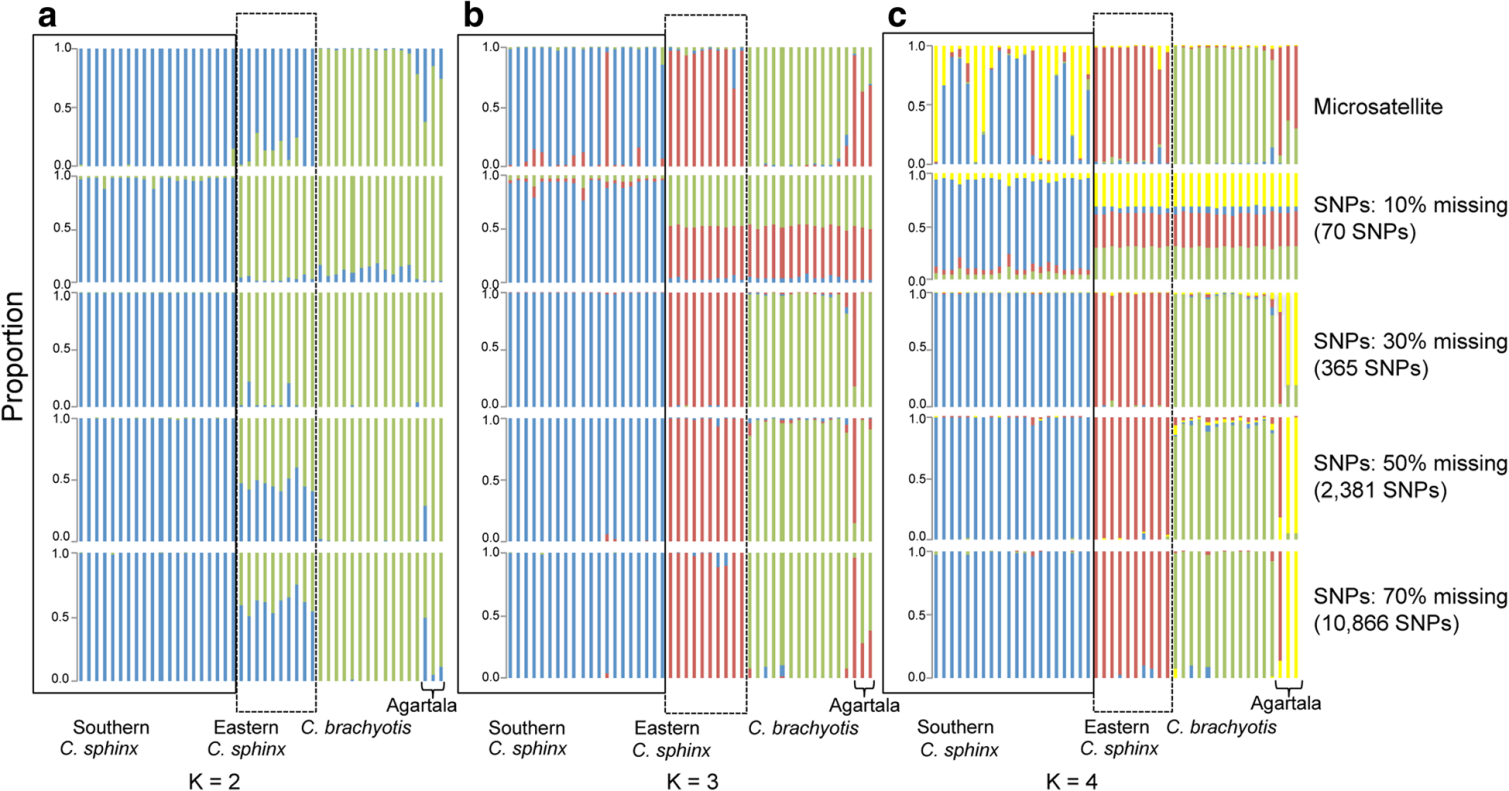
Barplots of the ancestry coefficient q of microsatellite (assignment with all 9 loci) and various SNP datasets at **a**) K = 2, **b**) K = 3 and **c**) K = 4. Intraspecific subdivisions within *C. sphinx* (eastern *C. sphinx* and southern *C. sphinx*) and the Agartala lineage were identified from the comparative analysis of SNP dataset and are specified accordingly

We used q value cutoffs based on previous trends obtained from the microsatellite dataset to denote pure and admixed categories in the assignment tests. We considered individuals with q values > 0.85 and < 0.15 as pure and individuals with q values from 0.15 to 0.85 as admixed. The most probable number of clusters was found to be 2. We compared trends across each K value for all four datasets and also compared between the Ks’ of each dataset. The results reveal that at K = 2, for all levels of missing data, the SNPs could distinguish between the species as well as retrieve intraspecific variation in agreement with microsatellite data. However, eastern Indian *C. sphinx* group (eastern *C. sphinx*, locations: 17, 18 and 19 in Fig. 1 and Additional file 1: Table S1) emerged with variable affinities and levels of purity (Fig. 4), as a distinct cluster from samples from southern and peninsular India *C. sphinx* group (southern *C.* sphinx, locations: 1, 2, 4, 6, 9, 10-15 in Fig. 1, Additional file 1: Table S1). Specifically, in the eastern *C. sphinx* group the genomic contribution of the *C. sphinx* cluster increased with a rise of missing data and the concomitant increase in the number of loci. In comparison, assignments with K = 3 apportioned the third genetic cluster to a homogeneous eastern *C. sphinx* group in most datasets (except for the analysis with the least number of loci; Fig. 4b). K = 4 revealed further substructuring in dividing out two individuals from Agartala (Agartala cluster, location: 19, Additional file 1: Table S1) as distinct, with increasing levels of purity as the number of loci increases (Fig. 4c). A third individual from Agartala emerged with possible admixed ancestry between the eastern *C. sphinx* cluster and the Agartala cluster. It has to be noted that allowing for more missing data in STACKS does not result in a significant increase in missing data (Additional file 3: Table S11), but rather in a pronounced increase in the number of loci available for analysis. Similar to the microsatellite dataset, we did not observe any biologically relevant clusters for K5 and K6 (data not shown).

We also used STRUCTURE to obtain net nucleotide distances between the four clusters at K = 4 for the 50 % missing dataset. We took an average of all ten iterations and observed that the Agartala cluster is almost equidistant from the eastern *C. sphinx* cluster and the *C. brachyotis* cluster and most distant from southern *C. sphinx* (Additional file 3: Table S12). Both clusters of *C. sphinx* were genetically very similar compared to the other clusters.

### Mitochondrial DNA-based phylogenetic reconstruction

**Fig. 5:**
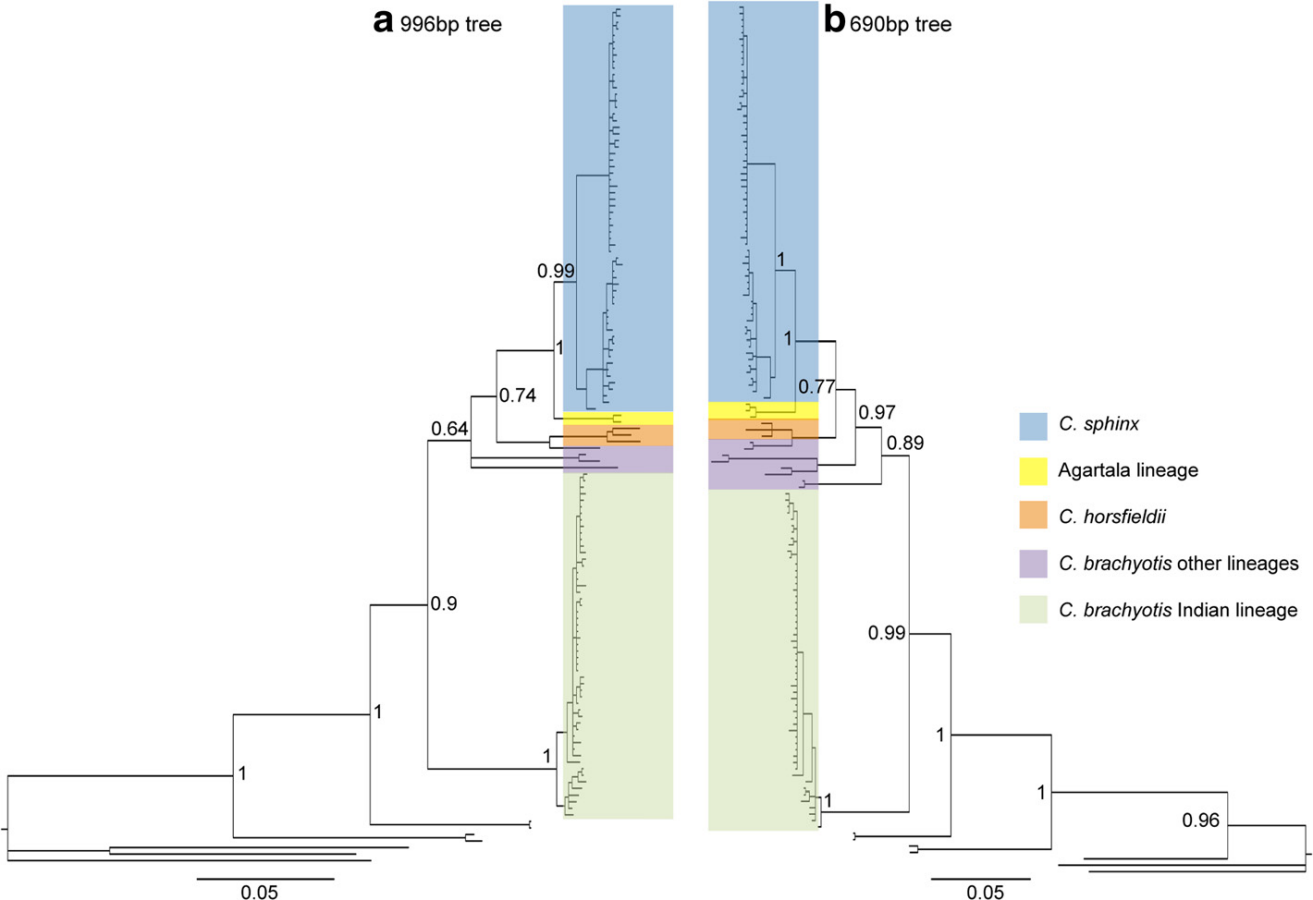
Phylogenetic tree of cyt*b* sequences **a**) 996 bp from 131 samples; **b**) 690 bp from 136 samples. The node labels represent posterior probability support for the respective node. The clade colored blue represents *C. sphinx*, yellow represents the Agartala lineage (in Fig. 5b, it also consists of a single sequence representing the *C. brachyotis* Myanmar lineage), orange represents *C. horsfieldii*, purple color represents all the other lineages of *C. brachyotis* and green represents *C. brachyotis* from India

In agreement with Campbell et al. [3], our cyt*b* tree supported monophyly of the Indian *C. brachyotis* lineage as well as the *C. sphinx* lineage (Fig. 5a and b). We also observed a general agreement between clade membership in the cyt*b* phylogeny, and clustering of microsatellites and SNPs in assignment tests. For all the putative admixed individuals, cyt*b* clade membership was identical to field identifications (Additional file 3: Table S10B). Individuals that remained unassigned in the field (Fig. 5a) were grouped in clades in agreement with the microsatellite and SNP data (Additional file 3: Table S10). We observed that the Indian *C. brachyotis* lineage is basal to all other sampled cynopterines (in agreement with Campbell et al. [3]). Genetic distance analysis (TrN + G substitution model) showed as much as a 9.4 % (0.094) divergence between the two species in agreement with Campbell et al. [3] (Additional file 9: Table S13). However, two individuals from the Agartala cluster (as based on SNP data) formed a separate monophyletic clade with high genetic divergence from the other *C. sphinx* lineages (0.04). Their average distance from the Indian *C. brachyotis* lineage (0.11) was also higher than that between the *C. sphinx* clade and the Indian *C. brachyotis* lineage (0.094). In the partial cyt*b* (690 bp) tree, which incorporated additional extra-limital samples, these individuals formed a separate clade with *C. brachyotis* from Myanmar, whereas *C. sphinx* from Myanmar was part of the *C. sphinx* clade (Fig. 5b). The putative admixed individual formed part of the *C. sphinx* clade and had a genetic distance of 0.019 with other *C. sphinx* and 0.052 with the Agartala cluster.

### Phylogenetic reconstruction from genome-wide data

**Fig. 6:**
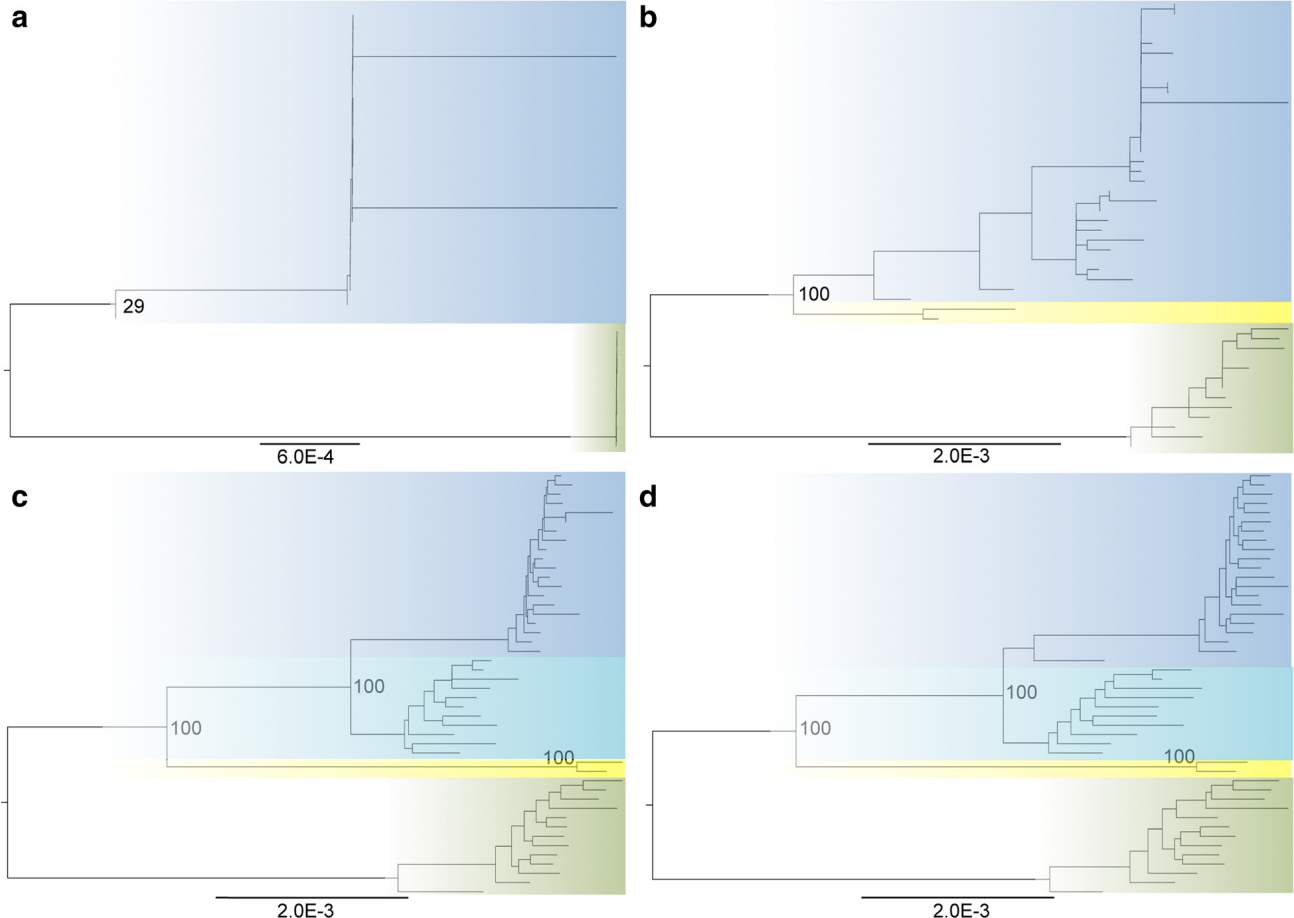
Midpoint rooted phylogenetic trees obtained from genome-wide sequence data. The clade colored blue represents *C. sphinx* and the clade colored green represents *C. brachyotis*. Different shades of blue represent different geographical clades within *C. sphinx*. The clade colored yellow represents the Agartala lineage. Figures **a**, **b**, **c** and **d** represents 10 %, 30 %, 50 % and 70 % missing data respectively

Phylogenetic reconstructions of the concatenated genome-wide sequence data with different levels of missing data unambiguously separated the species as distinct clades. Clade identities were identical to those observed in the cyt*b* phylogeny and genetic assignments with SNPs and microsatellites. Both species segregated as monophyletic clades when we allowed for 10 % missing data (Fig. 6). When we allowed more loci and sampled more regions of the genome, we observed that the two individuals representing Agartala cluster formed a monophyletic clade basal to all *C. sphinx* sequences in agreement with the cyt*b* tree (Fig. 6). Though the partial cyt*b* tree (which contained Myanmar material) classified Agartala individuals as part of the Myanmar *C. brachyotis* lineage, yet the absence of genomic data from the Myanmar *C. brachyotis* lineage restricts us from validating the congruence between nuclear genome phylogeny and mitochondrial genome phylogeny. Hence we categorize the Agartala cluster as the Agartala lineage. Further, with 50 % and 70 % missing data and more loci incorporated, the *C. sphinx* clade again subdivides into two reciprocally monophyletic clades, one representing the southern *C. sphinx* and the other representing the eastern *C. sphinx* in agreement with the genomic clusters (SNP data) identified using STRUCTURE analysis, but in contrast to the cyt*b* phylogeny, which lacked such resolution.

### Test for introgression

**Fig. 7:**
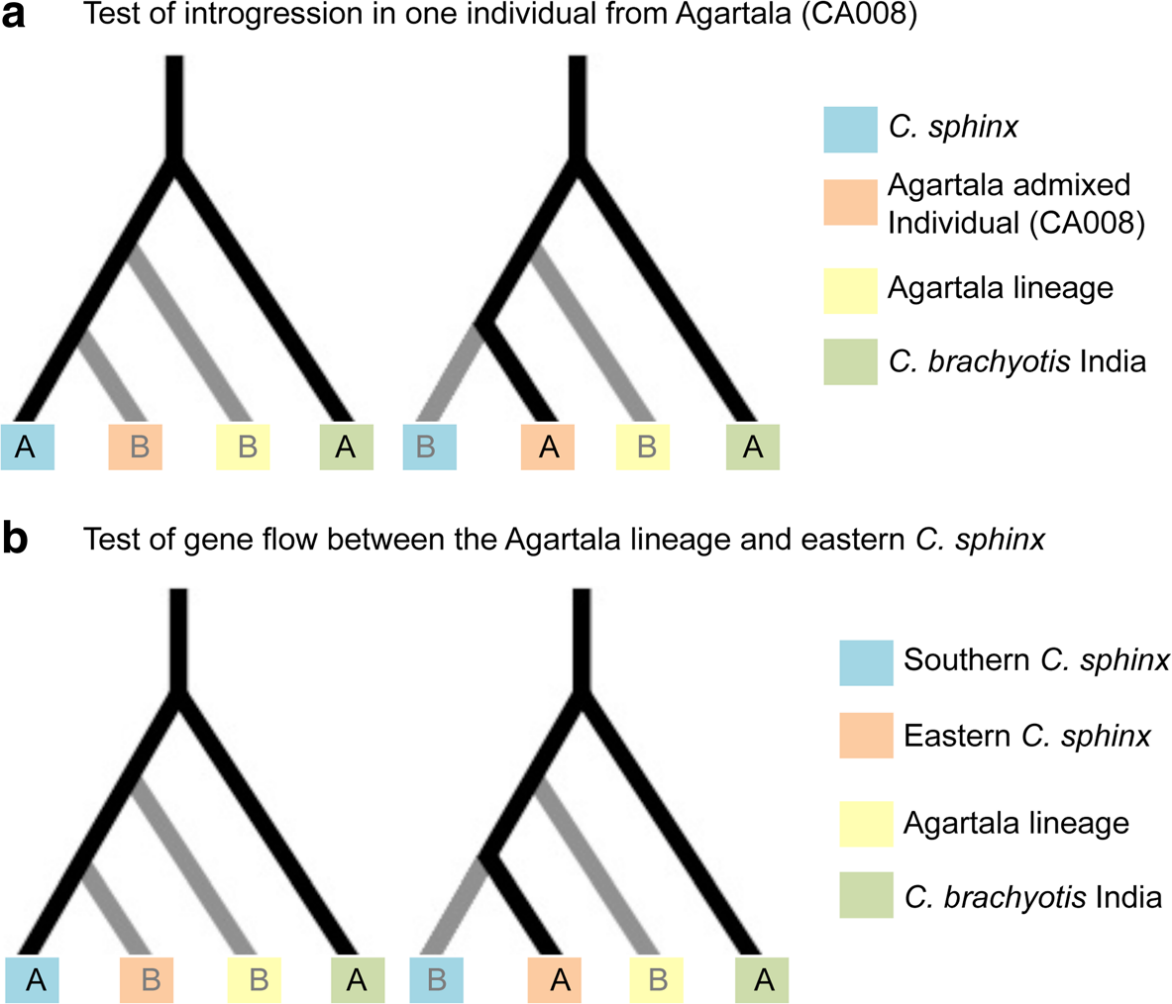
Graphical representation of clade arrangements for tests of introgression using Patterson’s D statistic: **a**) test of introgression in one individual from Agartala (CA008) and **b**) test of gene flow between the Agartala lineage and eastern *C. sphinx*

Using the 30 %, 50 % and 70 % missing datasets (the 10 % missing dataset did not contain any ABBA-BABA site), we performed tests of introgression for two different scenarios (Fig. 7) to investigate gene flow between *C. sphinx* and the Agartala lineage. We used Indian *C. brachyotis* as an outgroup. We first tested for possible introgression in one individual from Agartala (CA008), which was genomically a member of the eastern *C. sphinx* group with admixture from the Agartala lineage (represented by the other two Agartala individuals) (Figs. 4 and 7a). A significant difference between the number of ABBA and BABA sites suggested introgression (Table 1). For the second test we removed this introgressed individual and performed a test of gene flow between the Agartala lineage and the eastern *C. sphinx* cluster (Fig. 7b). We obtained similar results of significant differences between ABBA and BABA sites suggesting gene flow between these two lineages (Table 1).

**Table 1:** Summary of Patterson’ D test statistic. Significant values of D statistic are indicated in bold font

Dataset from pyRAD pipeline	Introgression in one individual from Agartala (CA008)		Gene flow between the Agartala lineage and eastern *C. sphinx*	
	D statistic	Z score	D statistic	Z score
10 % missing	NA	NA	NA	NA
30 % missing	-**0.41**	9.66	**0.33**	5.04
50 % missing	-**0.49**	94.15	**0.27**	34.42
70 % missing	-**0.47**	221.32	**0.15**	60.71

### Genetic data improves morphological classification

We used the cyt*b* clade identity as a grouping variable (n = 112). We performed a forward stepwise DA considering F to enter as 0.010, F to remove as 0.0 and the minimum tolerance as the default 0.01. We grouped our data into three categories, *C. sphinx*, *C. brachyotis* and the Agartala lineage. The best-fit model consisted of all three morphological variables with forearm length being the best explanatory variable (Additional file 3: Table S14). We further performed self-classification considering an equal probability of group membership. Overall 81 % of the samples (66.67 % of *C. sphinx* and 92.5 % of *C. brachyotis*) could be correctly identified based on morphology. The discriminant function had 100 % power to classify the two individuals from the Agartala lineage.

## Discussion

We investigated morphological and genetic differentiation of congeneric fruit bats and assessed the concordance between these two types of data. Our results reveal the importance of molecular markers, specifically genome-wide markers, in the discovery of cryptic diversity, leading to improved species identification and the documentation of significant range extensions. More importantly, we uncover an additional, hitherto unrecognized, cryptic lineage of *Cynopterus* coexisting with *C. sphinx* in northeastern India, and we identify individuals that bear the hallmark of introgression (based on genome-wide DNA) between these two lineages. These results provide the first detailed insights into the complicated patterns of differentiation among this cryptic bat radiation and significantly expand our knowledge of their biogeography and levels of reproductive isolation.

### Morphology-based identification is unreliable in contact zones

Available literature suggests considerable overlap in morphology between cynopterine species and advocates the use of genetic assignment tests for identification in such situations [18, 19]. Our study reiterates that a suite of external morphological characters widely used for species level identification may not be very informative, specifically in contact zones (Fig. 2d). The situation is particularly problematic in peninsular and southern India. Clinal morphological variation [52] within *C. sphinx* might be an important reason for such low classification power. Future sampling of both species across elevational gradients may reveal the extent of morphological similarity. Additionally, more sampling and cross validation is required to understand the difference between *C. sphinx* and the Agartala lineage. We propose that wherever possible cyt *b* sequencing or more specifically genome-wide SNP data should be generated to obtain species level assignment.

### Cryptic diversity of cynopterine bats in India

Genome-wide SNPs revealed discrete geographic lineages within *C. sphinx* in addition to a cryptic, hitherto unrecognized cynopterine lineage in northeastern India which coexists at least with *C. sphinx* (Fig. 4). Further, phylogenetic reconstructions reveal that the Agartala lineage is a sister species of *C. sphinx* and shares close genetic and phylogenetic proximity with the *C. brachyotis* Myanmar lineage (Fig. 5b). However, our understanding of the taxonomy of the Agartala lineage is limited due to the lack of a voucher specimen as well as due to the lack of comparative nuclear genomic material from *C. brachyotis* Myanmar. Future studies should address these issues and provide appropriate taxonomic revisions.

### Gene flow and introgression between cynopterine bats in India

Fruit bats of the genus *Cynopterus* often share large contact zones [3, 4, 20, 53]. We identified and assessed contact zones of *C. sphinx* and *C. brachyotis* in India and found no strong evidence of hybridization in these zones based on genome-wide SNPs; in contrast microsatellite data were inconclusive, probably based on their considerably lower level of resolution as compared to genome-wide SNPs (Figs. 3 and 4). Interestingly, SNP data revealed instances of gene flow between *C. sphinx* and the Agartala lineage. A comparison of mtDNA phylogeny, assignment tests and shared variability (ABBA-BABA tests) suggests at least one incidence of male-mediated introgression (CA008) from the Agartala lineage into the nuclear genome of *C. sphinx*. CA008 is an adult male with *C. sphinx* cyt*b* haplotype and a high nuclear contribution of *C. sphinx*. Additional tests further revealed hallmarks of introgression from the cryptic Agartala lineage into the eastern cluster of *C. sphinx*, including those individuals whose genome-wide SNP profile had appeared pure (Figs. 4 and 7b). The gene flow between these two lineages may be limited, therefore resulting in a level of introgression that is undetectable with genomic scans spanning thousands of loci and is only detected through specific tests that can distinguish between introgression and incomplete lineage sorting [49].

It is difficult to ascertain the relative extent of contemporary and historical gene flow between *C. sphinx* and the Agartala lineage as the test of shared variation lacks sufficient power [51]. The low sample size of the Agartala lineage within our dataset also prevents us from making a coalescent based model comparison to assess the pattern, extent and direction of gene flow between these two species. More extensive sampling in northeastern India and the Indo-Myanmar biodiversity hotspot is required to further unravel the evolutionary affinity of this lineage as well as systematically characterize patterns of gene flow between the two lineages.

Previous evolutionary inquiries into *C. sphinx* and other southeast Asian lineages of *C. brachyotis* could not obtain conclusive evidence of interspecies gene flow or the lack of it [53]. However in the light of the higher resolution provided by genome-wide data it will be interesting to revisit gene flow between various species of cynopterine fruit bats across their range in the Paleotropics, specifically since this group has experienced a very recent radiation and much of the interspecific relationships are polytomous and unresolved ([3, 16] and this study), thereby increasing the possibility of ancient gene flow during and/or after divergence. Additionally, the distributions of most of cynopterine species are nested within the broad distribution of *C. sphinx,* suggesting that the evolutionary history of *C. sphinx* may include complicated scenarios of admixture with different lineages. Our results using genome-wide DNA evidence lend support to previous case studies of interspecific hybridization in bats that have revealed gene flow in contact zones [54–57]. The lack of gene flow between *C. sphinx* and *C. brachyotis* in peninsular and southern India may be an artifact of insufficient sampling, as studies may miss rare admixture. However, lack of genomic data from an appropriate outgroup species in the current study also prevents us from further examination of low levels of admixture. More population and genomic sampling may provide additional insight into patterns of isolation between these two species.

### Genome-wide SNPs: more loci provide better resolution

One important trade-off while using a restriction enzyme based reduced representation library of genome-wide data is between the extent of missing data and the number of loci. Reducing missing data inevitably reduces the number of loci quite drastically, specifically when data from a single lane is analysed. However, recent studies have shown that mining more loci regardless of a significant amount of missing data may still provide more power to the data than sampling fewer loci with less missing data [10, 58]. A comparison of observations across various levels of missing data in our study also reveals that mining more loci with missing data rather than fewer loci that lack missing data can provide singnificantly more biologically relevant information in both population genomic and phylogenomic analyses (Figs. 4 and 6).

### Contrasting microsatellite/mitochondrial and genome-wide information

Although the microsatellite markers used in this study are too few in number and suffer from multiple drawbacks, they are able to identify purebred individuals. However, they suffer from low power and efficiency when identifying admixture (Additional file 3: Table S15). The ddRADseq data fare considerably better and reveal subtle intraspecific sub-structuring. Thus, future assignment studies may consider the generation and analyses of genome-wide loci as performed in this study. This is of particular advantage specifically because a few hundred individuals can be sequenced in a single lane. Mitochondrial cyt*b* data were also effective in assigning individuals to species level and can be used for initial identification purposes, but they are inherently problematic in the assignment of admixed individuals as they will only reveal information about the matrilineal ancestry.

## Conclusion

Our study uses thousands of genome-wide markers from natural populations of Old World fruit bats to address the complex evolutionary dynamics of a recent radiation. Our SNP data identified an unrecognized, cryptic lineage of cynopterine fruit bat in northeastern India, and provided evidence of admixture and introgression between *C. sphinx* and this cryptic lineage. Our results suggest caution when using standard external morphological traits in species identification within the cynopterine radiation, especially for the broadly distributed *C. sphinx,* and emphasizes the utility of genetic markers in species identification when morphology is inaccurate. The use of large number of genetic markers not only improves assignment of individuals at the species level, but also uncovers fine-scale genetic differentiation patterns which maybe particularly important when studying species with large distributional ranges. Using fewer genetic markers (microsatellites as well as SNPs) in such cases may lead to misinterpretation of intraspecific differentiation as gene flow.

### Availability of data and material

Datasets supporting the results of this article are available in GenBank (KF042154-KF042252 and KJ417498-KJ417512) and Sequence Read Archive (SRP047152). Microsatellite genotype data is available as Additional file 10.

## Additional files

Additional file 1: Table S1. Sampling details consisting of information about number of individuals typed for each marker. Populations with mutiple sampling points are represented by one representative coordinate. FA = forearm length, EL = ear length and TIB = tibia length. All morphological measurements are recorded in millimeters (mm). (XLSX 13 kb) Additional file 2:
**Supplementary methods and results.** (DOCX 111 kb) Additional file 3:
**Table S2.** Rounding error of each microsatellite locus, the proportion of missing data, number of alleles and allele size range of each microsatellite locus. bp = base pair. **Table S3.** Test for the saturation of phylogenetic signal in the mitochondrial DNA dataset. **Table S4.** Summary statistics of morphological variables. N = Number of samples; SD = Standard Deviation. **Table S5.** Tests to assess the presence of size dimorphism within each species. **Table S6.** Contribution of morphological variables in each PCA dimension. **Table S8.** Table summarizing the results of the test for neutrality. Values in bold indicate loci under selection. We used a 5 % false discovery rate (FDR) to determine loci under selection. The q value is an FDR analog of *p* value. **Table S9.** Ancestry coefficient (q value) values of genetically admixed individuals. **Table S10.** Details showing genetic affinities of A) morphological unidentified individuals in field and B) genetic intermediates identified using all nine loci. **Table S11.** Average heterozygosity and average missing data of various SNP datasets obtained from STACKS. **Table S12.** Net nucleotide distance between different genetic clusters (K = 4) of the genome-wide SNP data (50 % missing dataset). **Table S14.** Summary statistics obtained from discriminant function analysis [Step 3, number of variables in the model: 3; Wilks’ Lambda: 0.3 approx. F (6, 140) = 13.97, p value < 0.001]. **Table S15.** Power and accuracy of the STRUCTURE program in detecting purebreds and hybrids from simulated microsatellite datasets. SD = standard deviation; HP = hybrid proportion. (DOCX 116 kb) Additional file 4: Figure S1. FastQC report of the ddRAD run. (PDF 3067 kb) Additional file 5: Figure S2. Correlation circle of the continuous variables. (PDF 536 kb) Additional file 6: Table S7. Population wise assessments of null alleles and deviation from Hardy-Weinberg equilibrium. (XLS 26 kb) Additional file 7: Figure S3. STRUCTURE harvester results. A) Mean estimate of log likelihood (LnP(D)) of each K, B) Estimate of delta K. (PDF 306 kb) Additional file 8: Figure S4. Barplot of the ancestry coefficient q at K = 2, 3 and 4 of A) all nine loci and B) neutral loci. (PDF 751 kb) Additional file 9: Table S13. Net interspecies genetic distances of the cyt*b* dataset. All distances were calculated using the TrN + G model. (XLSX 38 kb) Additional file 10:
**Microsatellite genotypes used in this study.** (XLSX 73 kb)
