# Supplementary material for: Genome-wide data reveal cryptic diversity and genetic introgression in an Oriental cynopterine fruit bat radiation
Source: BMC Evol Biol. 2016 Feb 18;16:41. doi: 10.1186/s12862-016-0599-y (PMC4757986; doi:10.1186/s12862-016-0599-y)
Supplement: Additional file 2: — Supplementary methods and results. (DOCX 111 kb) [file 12862_2016_599_MOESM2_ESM.docx]

**METHODS**

***PCR conditions for microsatellite amplification:***

PCR conditions for the amplification of the nine microsatellite markers broadly followed Chattopadhyay *et al*. [1]. However, in addition to Ampli-Taq Gold DNA polymerase (Applied Biosystems, n = 266) we also used PCR Master mix (MM, Qiagen, n = 121) for PCR amplification in the present study. Concentration of primers and other reagents were same as described in Chattopadhyay *et al.* [1].

***Error rate and missing data calculation:***

We amplified ~ 5% (n = 20) of the data to test for consistency between Ampli-Taq Gold and PCR Mastermix. We also re-amplified ~ 5% (n = 20) of samples genotyped using MM to estimate the genotyping error rate for PCR Mastermix following Hoffman and Amos [2]. Error rates between MM and Ampli-Taq was 0.007, and within MM was 0.009. Amplification errors for Ampli-Taq have been reported to be below 0.01 by Garg *et al*. [3], using the same set of markers. We considered the error rate within MM as representative of the overall error rate. We repeated PCRs once if they failed to amplify. Further unsuccessful amplification was considered as missing data.

***Sequencing cytb:***

We used a suite of generic primers [4] as well as specific primers designed for this study, to amplify the entire gene. We designed specific primers (flanking forward primer: F2 [GTCATCATTATTCCCACATGGACTCT], flanking reverse primer: R3 [GGTTGCTCCCCTTTTCTGGTTTACA], internal forward primers: FP300 [TCCTACATCTACACAGAAACATG] and FP448 [CTCTCAGCAATTCCATATATCG]), to amplify the entire 1140 bases of the cyt*b* gene. We obtained 80 µl of PCR product for each individual and reagent concentrations per reaction were - 2.5 mM MgCl_2_, 0.35 μM of each primer (F2 and R3), 0.2 mM of dNTPs and 8units/100 μl of Taq polymerase. Volume of template DNA added during amplification depended on the extent of amplification in screening PCRs. We performed amplifications with initial denaturation at 95 ^0^C for 5 minutes followed by 40 cycles of denaturation at 95 ^0^C for 1 minute, primer annealing at 37 ^0^C to 50 ^0^C for 45 seconds and extension at 72 ^0^C for 1 minute 30 seconds. A final extension was performed at 72 ^0^C for 5 minutes. PCR products were run in 1.5% Agarose Gel, for two hours at 50 V, in 1XTAE (Tris Acetate EDTA) buffer. A 100 base pair DNA ladder (Fermentas: GeneRuler^TM^ 100 base pair Plus DNA Ladder) was run in each gel alongside the samples. Gels were visualized under ultraviolet illumination and desired fragments sliced. PCR products were purified through gel extraction protocol using the QIAquick gel extraction kit (Qiagen, Germany), following the manufacturer’s protocol, and samples were suspended in 30 μl MilliQ water (Millipore, USA). 2 μl of purified product per sample were electrophoresed alongside equal volume of DNA ladder to quantify the concentration of the purified product.

Sequencing runs were performed in ABI3100XL Sequencer. The cycle sequencing PCR consisted of initial denaturation at 96 ^0^C for 3 minutes, followed by 30 cycles of denaturation at 94 ^0^C for 10 seconds, annealing at 50 ^0^C for 5 seconds and extension at 60 ^0^C for 4 minutes. Final extension was performed at 72 ^0^C for 7 minutes.

Sequencing strategy typically involved obtaining overlapping forward and reverse sequences using the flanking primers. Conversely if there was no distinct zone of overlap then we used one or more internal primers to obtain the missing sequence. All chromatograms were manually edited and aligned using FinchTV (http://www.geospiza.com/Products/finchtv.shtml) and MEGA 5.0 [5]. Sequences from the overlapping fragments were assembled and checked for the presence of stop codons using the vertebrate mitochondrial codon table in MEGA 5.0 [5]. We further confirmed the affinity of each sequence with available cynopterine cyt *b* sequences through local sequence alignment (BLASTN) from NCBI.

We obtained summary statistics for the full-length sequences in DnaSP 5.10.1 [6] and the results are summarized in table S16.

***Analyses of microsatellite loci:***

*a) Allele number and allele size range:*

We only considered the genetically pure samples based on STRUCTURE assignment to determine the number of alleles and the allele size range for each microsatellite loci. The number of alleles per species per locus was calculated using Cervus 3.0 [7] and the allele size range was manually determined (Table S2). We only considered populations with greater than five individuals for the above analysis (n = 366).

*b) Test for homoplasy and ascertainment bias:*

We addressed the question of homoplasy and ascertainment bias by comparing allele size ranges between allopatric populations of the two species. To check for homoplasy we first compared the average allele size range of each species to the mean allele size range of both species [8]. We also sequenced two common alleles (one each from loci CSP7 and CSP9) that were shared across species. For each of these alleles, we sequenced one homozygous individual from the following categories: genetically pure *C. sphinx*, genetically pure *C. brachyotis* and genetically admixed. A pairwise Wilcoxon rank sum test with continuity correction was performed to test for ascertainment bias. Allele size range at each locus for both the species was considered for this test. We selected samples which were morphologically typical and had a q value greater than 0.9.

We did not observe any homoplasy or ascertainment bias within our dataset. Cumulative allele size range of both species (67.78) was considerably higher than that within each species (*C. sphinx* – 52.2, *C. brachyotis* – 57) suggesting lack of saturation in microsatellite size range. Similarly, we did not observe any significant difference in allele size length between both species (p value = 0.8125). However, on sequencing common alleles we observed identity by state in allele size due to presence of indels and not repeat similarity. In order to reduce bias related to homoplasy, we performed all microsatellite based analyses assuming infinite allele model (IAM).

*c) Test for Hardy-Weinberg equilibrium:*

We performed global tests of heterozygote deficiency to test for deviation from Hardy–Weinberg equilibrium (HWE) in Genepop version 4.2.1 [9] within each species. We performed 10,000 dememorisations, 100 batches and 5,000 iterations per batch to compute p values. We removed the putative genetic intermediates (n = 13, conservative estimate based on 0.9 cut off in STRUCTURE) based on initial results from the STRUCTURE run. Individuals were segregated into two species and further into populations (19 locations; *C. sphinx* = 12, *C. brachyotis* = 7) and each species was separately analyzed. We only considered populations with greater than five individuals for the above analysis (n = 366, number of *C. sphinx* individuals = 223, number of *C. brachyotis* individuals = 143, Table S7).

*d) Test for null alleles:*

We removed the putative genetic intermediates (n = 13) based on initial results from the STRUCTURE run and considered totally 366 samples for this analysis. Similar to the previous analysis individuals were segregated into two species and further into populations (19 locations; *C. sphinx* = 12, *C. brachyotis* = 7), each species was separately analyzed, and populations with greater than five individuals were considered (Table S7).

***Efficiency of genetic assignment using STRUCTURE:***

We followed Burgarella *et al*. [10] and tested the power of our microsatellite loci towards individual assignment in STRUCTURE and compared the efficiency of all nine loci to that of the neutral loci in identifying purebreds and admixed individuals. We used the pure individuals (q value > 0.90, following Vaha and Primmer, [11], Burgarella *et al*. [10]) of both species as representative of parental populations and generated 10,000 simulated individuals each for various admixed categories (hybrids, back crosses as well as hybrid F1 individuals) using Hybridlab 1.0 [12]. Hybridlab uses the observed allele frequencies and generates simulated genotypes assuming Hardy Weinberg Equilibrium. We further selected a subset of 150 individuals representative of each pure class and totally 20 admixed individuals (5 from each admixed class) through random sampling (without replacement) using PopTools 3.2.5 [13]. We performed separate STRUCTURE runs, 1) only with the pure individuals (300 samples) reflective of no admixture scenario and 2) with both pure (n = 300) and admixed individuals (n = 20) reflective of a rare admixture scenario. We created five such random datasets for both scenarios using PopTools to test the efficiency of the STRUCTURE program for our species. We implemented Structure runs considering K=2 (10 iterations) for all datasets. The proportion of admixed individuals was in accordance with the observed dataset. We analyzed the ancestry coefficients (q) of each individual to assess the performance of STRUCTURE in distinguishing genetically distinct clusters as well as pure and admixed individuals. We followed Burgarella *et al*. [10], and obtained estimates of the hybrid proportion (proportion of individuals in the sample identified as hybrid); efficiency (power to detect the true hybrid/purebred status of individuals - proportion of correctly identified individuals within each category); accuracy (number of correctly identified individuals of a category over total number of individuals assigned to that category); and type I error – proportion of wrongly identified as hybrids over total number of actual purebreds in the sample. We performed all analyses for all loci as well as for only the neutral loci.

We used a general cutoff of q value < 0.10 and > 0.90 for pure individuals and q value ≥ 0.10 and ≤ 0.90 for admixed individuals. Simulations revealed that the misassignment probability of purebreds is insignificant in our dataset, suggesting that these loci (both the entire set as well as the neutral subset) are robust in genetic assignment (Table S15). Within our dataset, STRUCTURE could assign purebreds with greater accuracy than admixed individuals under a rare admixture scenario (Table S15). We also obtained cutoff q values for two categories pure and admixed, from the simulated data. In the simulated data with all nine loci under the no admixture scenario, we observed two individuals with q values of 0.86 and 0.88 (simulated *C. sphinx*). All other individuals had q values above 0.90 and below 0.10. We thus used conservative cutoff values for pure individuals of > 0.85 and < 0.15 and for intermediates of ≤ 0.85 and ≥ 0.15. With simulated data comprising only the neutral loci, the accuracy in assigning purebreds was 98%, whereas the accuracy to assign admixed individuals was less than 70% (Table S15). Based on the q values of the simulated purebreds, we used cutoff values for pure individuals of > 0.70 and < 0.30 and for intermediates of ≤ 0.70 and ≥ 0.30.

***Phylogenetic analysis:***

We performed phylogenetic reconstruction of our dataset and also made an attempt to reconstruct phylogeny of the genus *Cynopterus* from available cyt *b* sequences. We specifically targeted sequences that might represent each genetic lineage reported by Campbell *et al*. [14]. We could retrieve full length sequences representing the Sunda lineage (GU724956), the Phillipines lineage (AB046320, AB046321) and the Forest lineage (GQ410210) of *C. brachyotis* group. We also downloaded partial sequences (more than 1kb) belonging to *C. sphinx* from South East Asia (FJ489964, FJ489958, JX283292, DQ445703, FJ489972) and full length *C. horsfieldii* sequences (EF201637, EF201639 and EF201643). Following Almeida *et al*. [15] we considered *Ptenochirus* as an outgroup to *Cynopterus*. We performed multiple sequence alignment allowing for gaps using ClustlW implemented in MEGA 5.0 [5]. *Ptenochirus jagori* (FJ218480 and GQ410211), *Pteropus vampyrus* (EF584230 and JN398212), *Rhinolophus ferrumequinum* (EU436673) *Hipposideros bicolor* (DQ054808) and *Megaderma lyra* (DQ888678) cyt*b* gene sequences were downloaded from the Genbank database and used as outgroups. After alignment and end pruning we could proceed with 996 bp for phylogenetic reconstruction.

Before choosing downloaded sequences as representatives of respective lineages, we aligned them with haplotypes generated by Campbell *et al.* [14] in MEGA [5]. We then obtained a neighbor-joining tree [5] and classified these sequences to genetic lineages following Campbell *et al.* [14] based on their phylogenetic affinity (monophyly with a known lineage) in the tree.

We performed model testing in jModeltest 0.1.1 [16] to obtain the best substitution model. Based on the least AICc value we further used HKY + gamma as the substitution model for our analysis (Table S17).

In addition to the above analysis, we also performed phylogenetic analysis with a shorter fragment of cyt*b* (690 bp) to accommodate other lineages of *C. brachyotis*. We repeated the above analysis along with the following sequences from Genbank (AY629000, *C. sphinx* Myanmar lineage; AY628945, *C. brachyotis* Myanmar lineage; AY628923, *C. brachyotis* Sunda lineage; AY628937 and AY628938 *C. brachyotis* Sulawesi lineage; and AY628966, *C. brachyotis* Forest lineage).

***Test for saturation of phylogenetic signal in cyt b:***

We also tested for homoplasy (saturation of phylogenetic information) within our dataset. Saturation of substitutions represents similarity based on identity by state rather than identity by descent and often proves problematic for phylogenies with deep branch lengths. We followed Song *et al*. [17] and performed a test to evaluate the extent of saturation of substitutions within the cyt*b* gene across all sequences used for phylogenetic analysis. We obtained Xia’s saturation index (Iss) in DMABE [18] and compared it to the critical values assuming symmetric topology (Iss.cSym). We assessed the p values to test for statistical significance. We divided the dataset into codon based partitions (first and second codon forms the first partition and third codon forms the second partition) and assessed levels of saturation in both partitions separately (Table S3).

***ddRADseq library preparation***

Library preparation followed Peterson *et al*. [19]. In brief, we performed restriction digestion for 3 hrs at 37 ^0^C following the manufacturer’s protocol. Samples were further purified using Ampure beads (Beckmann and Coulter) and quantified using Qubit (Invitrogen). We further ligated the cleaned products with P2 adapters at 16 ^0^C for 13 hrs. Samples of equal concentration were pooled into five libraries and size selected using Pippin Prep (Sage Science) (340 to 412 bp). We further performed streptavidin based cleanup to remove fragments which lacked the P2 adapter, amplified the fragments (12 cycles) and subsequently cleaned them using Ampure beads. Library validation was done with bioanalyzer profiles (Agilent Technologies) and qPCR.

**REFERENCES**

1. Chattopadhyay B, Garg KM, Doss PS, Ramakrishnan U, Kandula S. Molecular genetic perspective of group-living in a polygynous fruit bat, *Cynopterus sphinx.* Mamm Biol. 2011; 76:290-294.

2. Hoffman J, Amos W. Microsatellite genotyping errors: detection approaches, common sources and consequences for paternal exclusion. Mol Ecol. 2005; 14:599-612.

3. Garg KM, Chattopadhyay B, Doss D, A K Vinoth, Kandula S, Ramakrishnana U. Promiscuous mating in the harem-roosting fruit bat, *Cynopterus sphinx*. Mol Ecol. 2012; 21:4093-4105.

4. Irwin DM, Kocher TD, Wilson AC. Evolution of the cytochrome b gene of mammals. J Mol Evol. 1991; 32:128-144.

5. Tamura K, Peterson D, Peterson N, Stecher G, Nei M, Kumar S. MEGA5: molecular evolutionary genetics analysis using maximum likelihood, evolutionary distance, and maximum parsimony methods. Mol Biol Evol. 2011; 28:2731-2739.

6. Librado P, Rozas J. DnaSP v5: a software for comprehensive analysis of DNA polymorphism data. Bioinformatics. 2009; 25:1451-1452.

7. Kalinowski ST, Taper ML, Marshall TC. Revising how the computer program CERVUS accommodates genotyping error increases success in paternity assignment. Mol Ecol. 2007; 16:1099-1106.

8. Ochieng JW, Steane DA, Ladiges PY, Baverstock PR, Henry RJ, Shepherd M. Microsatellites retain phylogenetic signals across genera in eucalypts (Myrtaceae). Genet Mol Biol. 2007; 30:1125-1134.

9. Rousset F. Genepop’007: a complete re‐implementation of the Genepop software for Windows and Linux. Mol Ecol Res. 2008; 8:103-106.

10. Burgarella C, Lorenzo Z, Jabbour-Zahab R, Lumaret R, Guichoux E, Petit RJ et al. Detection of hybrids in nature: application to oaks (*Quercus suber* and *Q. ilex*). Heredity. 2009; 102:442-452.

11. Vaha J-P, Primmer CR. Efficiency of model-based Bayesian methods for detecting hybrid individuals under different hybridization scenarios and with different numbers of loci. Mol Ecol. 2006; 15:63-72.

12. Nielsen EE, Bach LA, Kotlicki P. HYBRIDLAB (version 1.0): a program for generating simulated hybrids from population samples. Mol Ecol Notes. 2006; 6:971-973.

13. Hood G. PopTools version 3.0. 6. 2008. URL http://www cse csiro au/poptools.

14. Campbell P, Schneider CJ, Adnan AM, Zubaid A, Kunz TH. Phylogeny and phylogeography of Old World fruit bats in the *Cynopterus brachyotis* complex. Mol Phylogenet Evol. 2004; 33:764-781.

15. Almeida FC, Giannini NP, DeSalle R, Simmons NB. Evolutionary relationships of the old world fruit bats (Chiroptera, Pteropodidae): Another star phylogeny? BMC Evol Biol. 2011; 11:281.

16. Posada D. jModelTest: phylogenetic model averaging. Mol Biol Evol. 2008; 25:1253-1256.

17. Song N, Liang A-P, Bu C-P. A Molecular phylogeny of Hemiptera inferred from mitochondrial genome sequences. PloS One. 2012; 7:e48778.

18. Xia X, Xie Z. DAMBE: software package for data analysis in molecular biology and evolution. J Hered. 2001; 92:371-373.

19. Peterson BK, Weber JN, Kay EH, Fisher HS, Hoekstra HE. Double digest RADseq: an inexpensive method for de novo SNP discovery and genotyping in model and non-model species. PLOS One. 2012; 7:e37135.
