# Supplementary material for: Genome-wide data reveal cryptic diversity and genetic introgression in an Oriental cynopterine fruit bat radiation
Source: BMC Evol Biol. 2016 Feb 18;16:41. doi: 10.1186/s12862-016-0599-y (PMC4757986; doi:10.1186/s12862-016-0599-y)
Supplement: Additional file 3: — Table S2. Rounding error of each microsatellite locus, the proportion of missing data, number of alleles and allele size range of each microsatellite locus. bp = base pair. Table S3. Test for the saturation of phylogenetic signal in the mitochondrial DNA dataset. Table S4. Summary statistics of morphological variables. N = Number of samples; SD = Standard Deviation. Table S5. Tests to assess the presence of size dimorphism within each species. Table S6. Contribution of morphological variables in each PCA dimension. Table S8. Table summarizing the results of the test for neutrality. Values in bold indicate loci under selection. We used a 5 % false discovery rate (FDR) to determine loci under selection. The q value is an FDR analog of p value. Table S9. Ancestry coefficient (q value) values of genetically admixed individuals. Table S10. Details showing genetic affinities of A) morphological unidentified individuals in field and B) genetic intermediates identified using all nine loci. Table S11. Average heterozygosity and average missing data of various SNP datasets obtained from STACKS. Table S12. Net nucleotide distance between different genetic clusters (K = 4) of the genome-wide SNP data (50 % missing dataset). Table S14. Summary statistics obtained from discriminant function analysis [Step 3, number of variables in the model: 3; Wilks’ Lambda: 0.3 approx. F (6, 140) = 13.97, p value < 0.001]. Table S15. Power and accuracy of the STRUCTURE program in detecting purebreds and hybrids from simulated microsatellite datasets. SD = standard deviation; HP = hybrid proportion. (DOCX 116 kb) [file 12862_2016_599_MOESM3_ESM.docx]

Table S1: Sampling details consisting of information about number of individuals typed for each marker. Populations with mutiple sampling points are represented by one representative coordinate. FA = forearm length, EL = ear length and TIB = tibia length. All morphological measurements are recorded in millimeters (mm).

Table S2: Rounding error of each microsatellite locus, the proportion of missing data, number of alleles and allele size range of each microsatellite locus. bp = base pair.

| Locus | Rounding error rate (bp) | Proportion of missing data | Number of alleles | | Allele size range (bp) | |
| --- | --- | --- | --- | --- | --- | --- |
|  |  |  | *C. sphinx* | *C. brachyotis* | *C. sphinx* | *C. brachyotis* |
| CSP1 | 0.61 | 0.003 | 15 | 14 | 182-224 | 167-230 |
| CSP2 | 0.38 | 0.005 | 9 | 6 | 108-132 | 93-135 |
| CSP3 | 0.26 | 0.00 | 11 | 10 | 91-124 | 91-118 |
| CSP4 | 0.26 | 0.00 | 8 | 8 | 121-161 | 129-161 |
| CSP5 | 0.6 | 0.00 | 17 | 15 | 127-195 | 111-183 |
| CSP6 | 0.57 | 0.00 | 13 | 14 | 119-179 | 115-195 |
| CSP7 | 0.8 | 0.005 | 15 | 14 | 218-278 | 218-270 |
| CSP8 | 0.64 | 0.018 | 18 | 34 | 144-216 | 144-284 |
| CSP9 | 0.47 | 0.005 | 8 | 3 | 273-301 | 269-277 |

Table S3: Test for the saturation of phylogenetic signal in the mitochondrial DNA dataset.

| Number of OTU | Iss | Iss.cSym | p value |
| --- | --- | --- | --- |
| Codon position one and two | | | |
| 4 | 0.044 | 0.805 | < 0.001 |
| 8 | 0.043 | 0.766 | < 0.001 |
| 16 | 0.045 | 0.745 | < 0.001 |
| 32 | 0.047 | 0.719 | < 0.001 |
| Codon position three | | | |
| 4 | 0.515 | 0.783 | < 0.001 |
| 8 | 0.495 | 0.736 | < 0.001 |
| 16 | 0.490 | 0.686 | 0.6619 |
| 32 | 0.478 | 0.685 | < 0.001 |

Table S4: Summary statistics of morphological variables. N= Number of samples; SD= Standard Deviation.

|  | Morphological variable | N | Mean (mm)(±SD) | Range (mm) |
| --- | --- | --- | --- | --- |
| *C. sphinx* (allopatry) | Forearm length | 183 | 69.6 (2.5) | 63.4 - 77.4 |
|  | Tibia length | 105 | 27.7 (1.5) | 24.0 - 31.4 |
|  | Ear length | 100 | 19.2 (1.7) | 15.8 - 24.0 |
| *C. sphinx* (contact zone) | Forearm length | 32 | 67.3 (1.9) | 64.6 - 70.6 |
|  | Tibia length | 18 | 26.0 (1.3) | 24.0 - 28.8 |
|  | Ear length | 21 | 18.3 (1.2) | 15.9 - 20.3 |
| *C. brachyotis* (allopatry) | Forearm length | 66 | 62.1 (3.0) | 50.8 - 67.2 |
|  | Tibia length | 49 | 24.0 (1.0) | 21.7 - 26.0 |
|  | Ear length | 82 | 16.1 (1.3) | 13.2 - 19.0 |
| *C. brachyotis*  (contact zone) | Forearm length | 66 | 63.4 (2.2) | 59.0 - 70.1 |
|  | Tibia length | 38 | 24.1 (0.9) | 22.0 - 26.1 |
|  | Ear length | 49 | 15.9 (1.2) | 13.5 - 18.5 |
| Unclassified | Forearm length | 27 | 64.9 (3.8) | 57.6 – 72.3 |
|  | Tibia length | 23 | 25.0 (2.1) | 20.0 - 28.4 |
|  | Ear length | 26 | 16.5 (1.8) | 13.5 - 22.5 |

Table S5: Tests to assess the presence of size dimorphism within each species.

| *C. sphinx* | | | | |
| --- | --- | --- | --- | --- |
| Morphological variable | Test | p value | No. of males | No. of females |
| Forearm | Welch Two Sample t-test | 0.97 | 94 | 121 |
| Tibia | Welch Two Sample t-test | 0.26 | 54 | 69 |
| Ear length | Wilcoxon rank sum test with continuity correction | 0.11 | 52 | 69 |
| *C. brachyotis* | | | | |
| Forearm | Wilcoxon rank sum test with continuity correction | 0.36 | 70 | 63 |
| Tibia | Welch Two Sample t-test | 0.38 | 46 | 42 |
| Ear length | Welch Two Sample t-test | 0.97 | 51 | 48 |

Table S6: Contribution of morphological variables in each PCA dimension.

| Variable | Dimension 1 | Dimension 2 |
| --- | --- | --- |
| Forearm length | 0.943 | -0.213 |
| Ear length | 0.826 | 0.564 |
| Tibia length | 0.928 | -0.285 |

Table S7: Population wise assessments of null alleles and deviation from Hardy-Weinberg equilibrium. (Excel sheet)

Table S8: Table summarizing the results of the test for neutrality. Values in bold indicate loci under selection. We used a 5% false discovery rate (FDR) to determine loci under selection. The q value is an FDR analog of p value.

| Locus | q value | Alpha | F_ST_ |
| --- | --- | --- | --- |
| CSP1 | 0.41 | 0.001 | 0.11 |
| **CSP2** | **0.0** | **1.274** | **0.32** |
| CSP3 | 0.33 | -0.013 | 0.11 |
| CSP4 | 0.13 | -0.639 | 0.08 |
| CSP5 | 0.25 | 0.027 | 0.12 |
| CSP6 | 0.05 | -0.882 | 0.06 |
| **CSP7** | **0.0** | **-2.026** | **0.02** |
| **CSP8** | **0.0** | **-1.486** | **0.03** |
| **CSP9** | **0.0** | **1.263** | **0.32** |

Table S9: Ancestry coefficient (q value) values of genetically admixed individuals.

| Genetic intermediates based on STRUCTURE | q value estimated from STRUCTURE for K = 2 | |
| --- | --- | --- |
|  | All 9 loci | Only neutral loci (removing loci under selection: CSP 2,7,8 and 9) |
| 1CS | 0.716 | 0.471 |
| VSP2 | 0.758 | - |
| VSP14 | 0.781 | - |
| CA002 | 0.151 | 0.436 |
| CA005 | 0.260 | - |
| CA008 | 0.621 | - |
| 0007 | 0.218 | 0.301 |
| CbYer7 | 0.844 | 0.585 |
| CSE18 | - | 0.312 |
| CSM9 | - | 0.332 |
| CBKM42 | - | 0.655 |
| CBY8 | - | 0.446 |

Table S10: Details showing genetic affinities of A) morphological unidentified individuals in field and B) genetic intermediates identified using all nine loci.

Table S10A

| Intermediate sample | mtDNA clade | Nuclear DNA cluster | q value |
| --- | --- | --- | --- |
| 0110 | *C. brachyotis* | *C. brachyotis* | 0.00401 |
| CIB12 | *C. sphinx* | *C. sphinx* | 0.99747 |
| CIB13 | *C. sphinx* | *C. sphinx* | 0.99462 |
| CIB14 | *C. sphinx* | *C. sphinx* | 0.99496 |
| CIB16 | *C. sphinx* | *C. sphinx* | 0.99374 |
| CIC02 | *C. sphinx* | *C. sphinx* | 0.99744 |
| CIY19 | *C. brachyotis* | *C. brachyotis* | 0.0045 |
| CIY24 | *C. brachyotis* | *C. brachyotis* | 0.00575 |
| CIY28 | *C. brachyotis* | *C. brachyotis* | 0.00681 |
| CIT07 | *C. sphinx* | *C. sphinx* | 0.99476 |
| CIY8 | *C. brachyotis* | *C. brachyotis* | 0.03787 |
| CIV08 | *C. sphinx* | *C. sphinx* | 0.99268 |
| CIHW 17 | *C. brachyotis* | *C. brachyotis* | 0.00572 |
| CITS2 | *C. brachyotis* | *C. brachyotis* | 0.00553 |
| CITS3 | *C. brachyotis* | *C. brachyotis* | 0.0102 |
| CITS4 | *C. brachyotis* | *C. brachyotis* | 0.0028 |
| CITS5 | *C. brachyotis* | *C. brachyotis* | 0.00441 |
| CITS10 | *C. brachyotis* | *C. brachyotis* | 0.00363 |
| CIKM27 | *C. brachyotis* | *C. brachyotis* | 0.00355 |
| CIKM31 | *C. brachyotis* | *C. brachyotis* | 0.00388 |
| CIKM36 | *C. brachyotis* | *C. brachyotis* | 0.00293 |
| CIKM47 | *C. sphinx* | *C. sphinx* | 0.99314 |
| CIKM50 | *C. brachyotis* | *C. brachyotis* | 0.00349 |
| CIKM55 | *C. brachyotis* | *C. brachyotis* | 0.00405 |
| CIKM57 | *C. brachyotis* | *C. brachyotis* | 0.00341 |
| CIKM58 | *C. brachyotis* | *C. brachyotis* | 0.00537 |
| CIKM60 | *C. brachyotis* | *C. brachyotis* | 0.00451 |

Table S10B

| Sample | Average q value | cyt*b* |
| --- | --- | --- |
| In allopatry | | |
| 1CS | 0.71586 | *C. sphinx* |
| VSP2 | 0.75748 | *C. sphinx* |
| VSP14 | 0.781 | *C. sphinx* |
| CA002 | 0.15137 | *C. sphinx* |
| CA005 | 0.25957 | *C. sphinx* |
| CA008 | 0.62099 | *C. sphinx* |
| OOO7 | 0.21821 | *C. brachyotis* |
| In contact zone | | |
| CbYer7 | 0.84428 | *C. sphinx* |

Table S11: Average heterozygosity and average missing data of various SNP datasets obtained from STACKS.

| Data set | Average heterozygosity | Average percentage of missing data |
| --- | --- | --- |
| 10% data set | 0.35 | 64.64 |
| 30% data set | 0.22 | 56.79 |
| 50% data set | 0.14 | 62.02 |
| 70% data set | 0.24 | 70.81 |

Table S12: Net nucleotide distance between different genetic clusters (K= 4) of the genome-wide SNP data (50% missing dataset).

|  | Southern *C. sphinx* | Eastern *C. sphinx* | Agartala lineage | *C. brachyotis* |
| --- | --- | --- | --- | --- |
| Southern *C. sphinx* | * |  |  |  |
| Eastern *C. sphinx* | 0.069 | * |  |  |
| Agartala lineage | 0.156 | 0.124 | * |  |
| *C. brachyotis* | 0.125 | 0.101 | 0.123 | * |

Table S13: Net interspecies genetic distances of the cyt*b* dataset. All distances were calculated using the TrN+G model. (Excel sheet)

Table S14: Summary statistics obtained from discriminant function analysis [Step 3, number of variables in the model: 3; Wilks’ Lambda: 0.3 approx. F (6, 140) = 13.97, p value < 0.001].

| Variable | Wilks'  Lambda | Partial  Lambda | F-remove  (2,70) | p value | Tolerance | 1-Tolerance  (R-Square) | Standardized coefficients | |
| --- | --- | --- | --- | --- | --- | --- | --- | --- |
|  |  |  |  |  |  |  | Root 1 | Root 2 |
| Forearm length | 0.52 | 0.75 | 11.55 | < 0.001 | 0.33 | 0.67 | 1.13 | -0.05 |
| Ear length | 0.42 | 0.93 | 2.80 | 0.07 | 0.87 | 0.13 | 0.33 | 0.74 |
| Tibia length | 0.41 | 0.95 | 1.70 | 0.19 | 0.35 | 0.65 | -0.43 | -0.78 |

Table S15: Power and accuracy of the STRUCTURE program in detecting purebreds and hybrids from simulated microsatellite datasets. SD = standard deviation; HP = hybrid proportion.

| Percentage of simulated hybrids | No. of repetitions | No. of hybrids in the sample | Mean no. of hybrids (SD) | Estimated HP | Power | | Accuracy | | Type I error |
| --- | --- | --- | --- | --- | --- | --- | --- | --- | --- |
|  |  |  |  |  | Hybrid | Purebreds | Hybrid | Purebreds |  |
| All 9 loci | | | | | | | | | |
| 0 | 5 | 0 | 0.4 (0.55) | 0.001 | - | 0.999 | - | 0.999 | 0.001 |
| 6 | 5 | 20 | 17.2 (2.86) | 0.054 | 0.82 | 0.997 | 0.958 | 0.988 | 0.003 |
| Neutral loci | | | | | | | | | |
| 0 | 5 | 0 | 1.4 (1.52) | 0.005 | - | 0.984 | - | 0.984 | 0.016 |
| 6 | 5 | 20 | 17.8 (3.49) | 0.056 | 0.64 | 0.981 | 0.695 | 0.976 | 0.019 |

Table S16: Summary statistics of the mitochondrial dataset (cyt*b*, n = 1140bp).

| Comparison | Number of haplotypes | Haplotype diversity | Nucleotide diversity | Average number of nucleotide differences |
| --- | --- | --- | --- | --- |
| Within *C. sphinx* (n = 59) | 44 | 0.983 | 0.015 | 17.62 |
| Within *C. brachyotis*  (n = 53) | 39 | 0.970 | 0.006 | 7.07 |
| Entire dataset consisting of both *C. sphinx* and *C. brachyotis* (n = 112) | 83 | 0.989 | 0.051 | 57.77 |

Table S17: Nucleotide substitution model comparison and selection of the best substitution model in jModeltest. AICc denotes corrected Akaike information correction values; *∆* AICc values denote differences in corresponding AICc values.

| Model | AICc | *∆* AICc |
| --- | --- | --- |
| TrN+G | 13501.81 | 0 |
| HKY+G | 13502.71 | 0.90 |
| HKY+I+G | 13504.7 | 2.56 |
| TIM2+G | 13505.22 | 3.41 |
| TIM3+G | 13505.48 | 3.67 |
| TPM1uf+I+G | 13505.50 | 3.70 |
| TPM1uf+G | 13506.16 | 4.36 |
| TPM2uf+G | 13506.42 | 4.62 |
| TIM1+G | 13507.15 | 5.35 |
| TPM2uf+I+G | 13507.72 | 5.92 |
