# Supplementary figures and images for: Genome-wide data reveal cryptic diversity and genetic introgression in an Oriental cynopterine fruit bat radiation
Source: BMC Evol Biol. 2016 Feb 18;16:41. doi: 10.1186/s12862-016-0599-y (PMC4757986; doi:10.1186/s12862-016-0599-y)

Quality scores across all bases (Sanger / Illumina 1.9 encoding)

Phred score

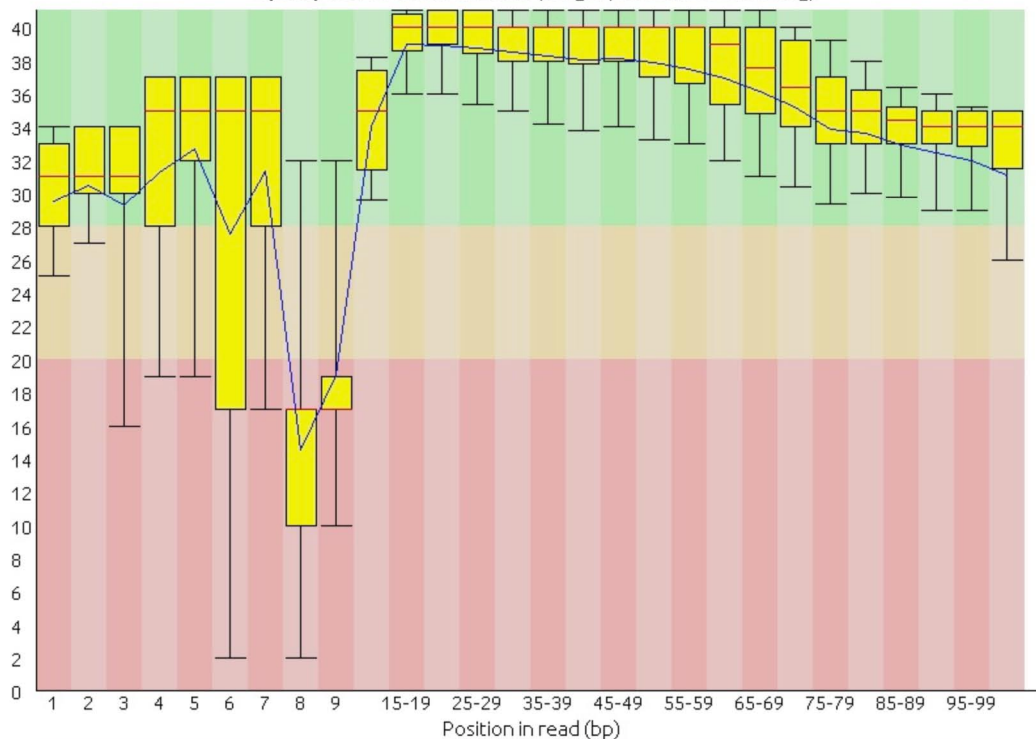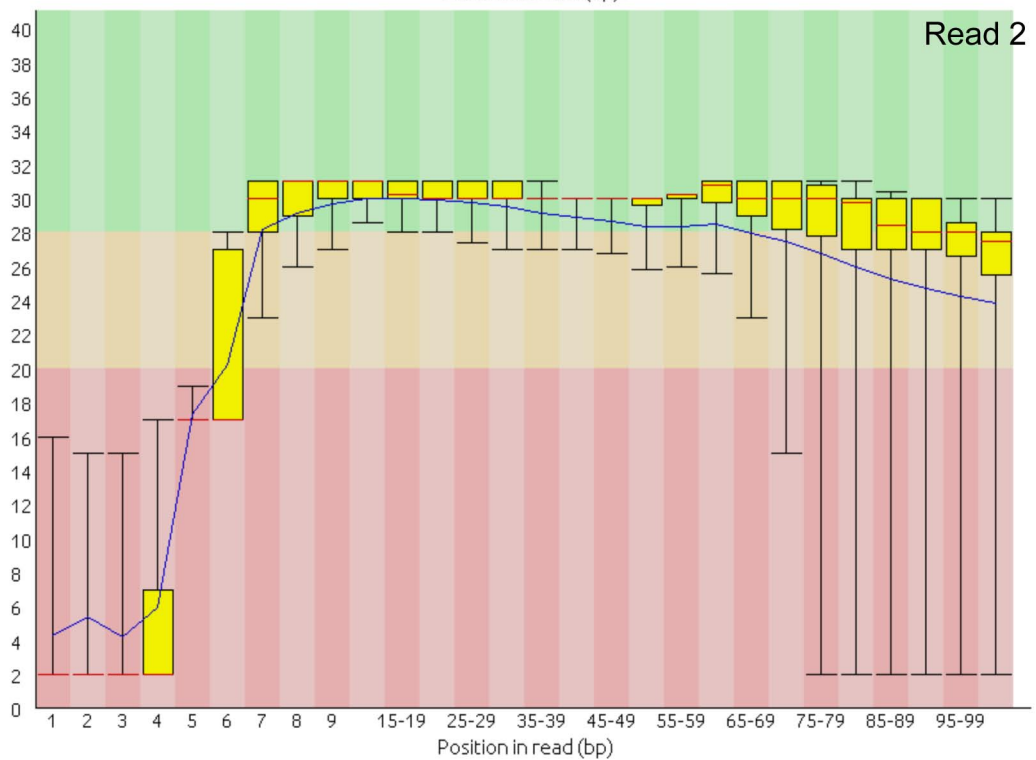

Supplement: Additional file 4: Figure S1. — FastQC report of the ddRAD run. (PDF 3067 kb) [file 12862_2016_599_MOESM4_ESM.pdf]

# Variables factor map (PCA)

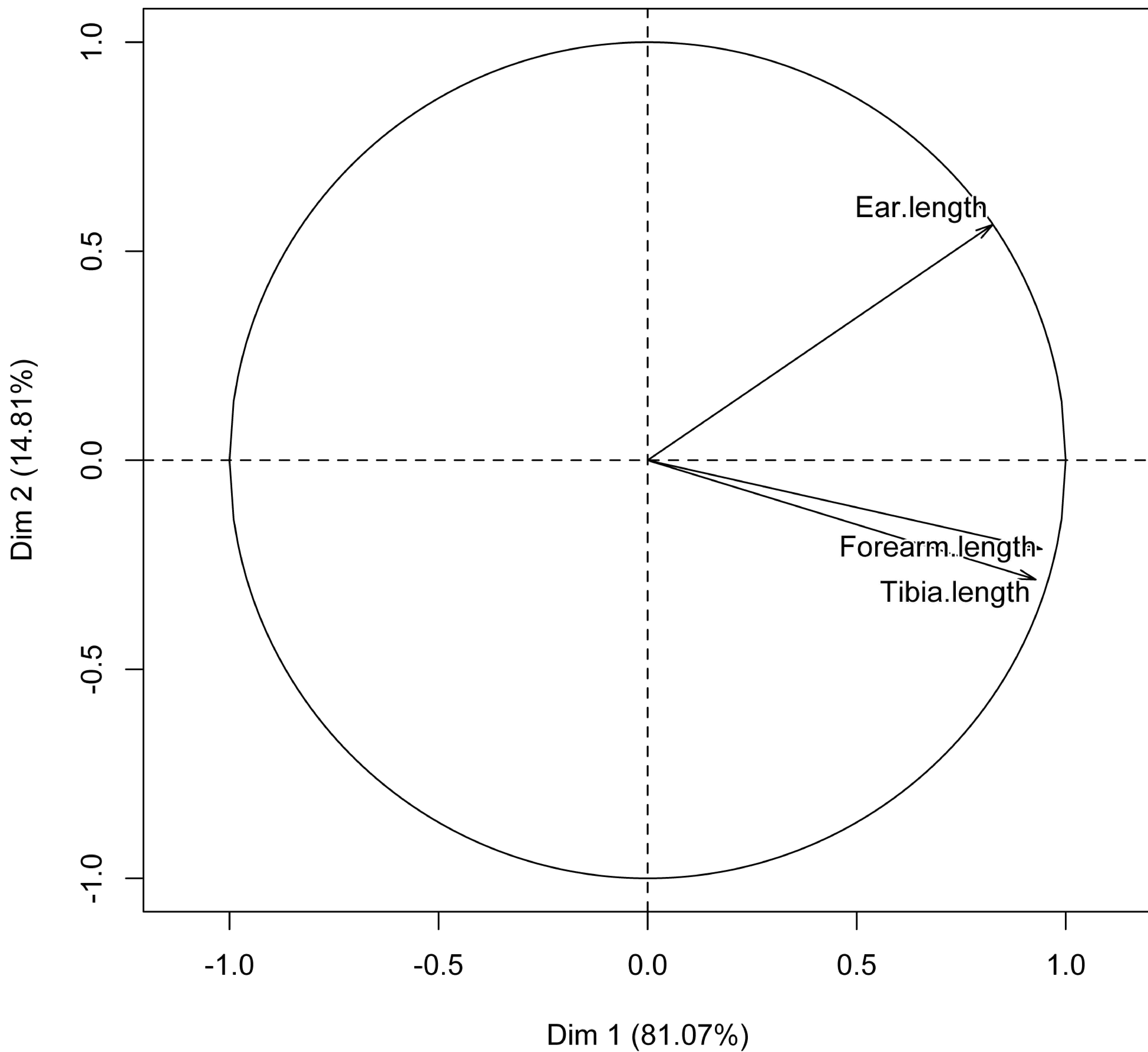

Supplement: Additional file 5: Figure S2. — Correlation circle of the continuous variables. (PDF 536 kb) [file 12862_2016_599_MOESM5_ESM.pdf]

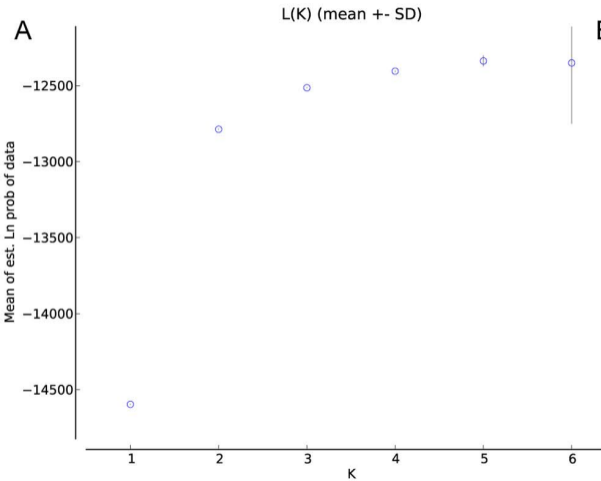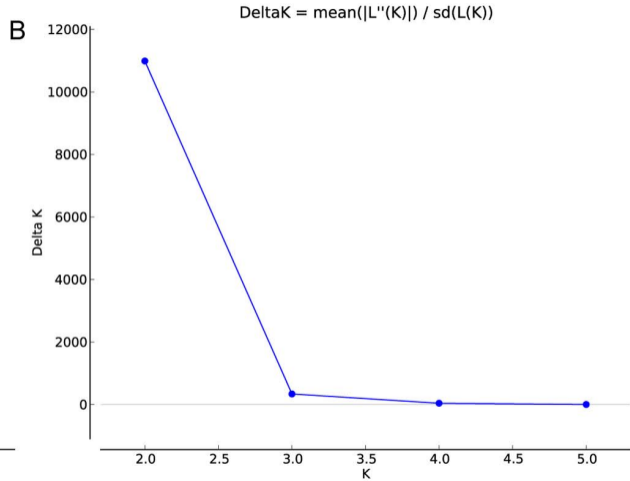

Supplement: Additional file 7: Figure S3. — STRUCTURE harvester results. A) Mean estimate of log likelihood (LnP(D)) of each K, B) Estimate of delta K. (PDF 306 kb) [file 12862_2016_599_MOESM7_ESM.pdf]

A) All nine loci

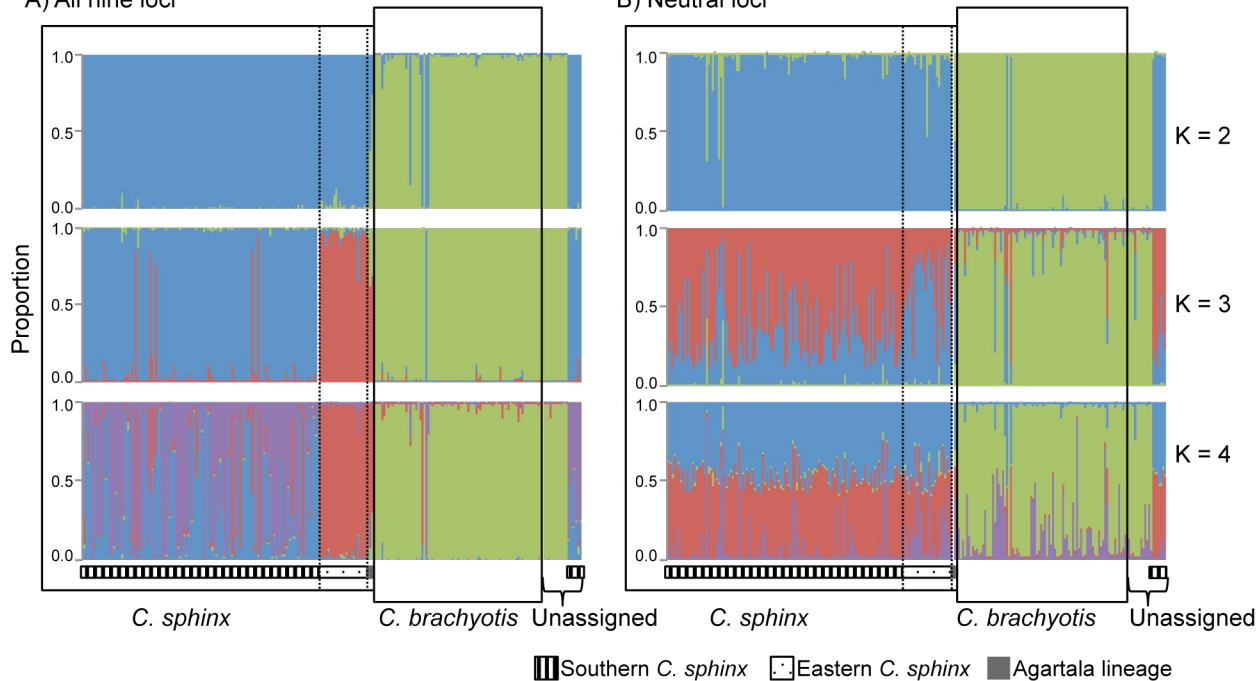

B) Neutral loci

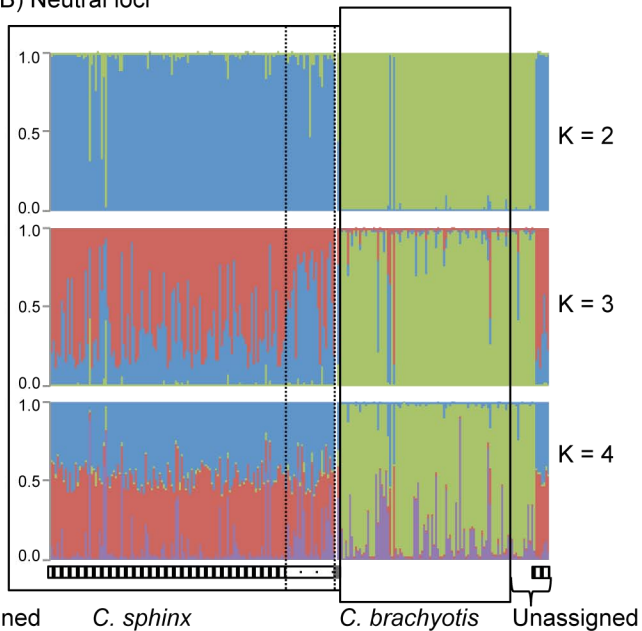

Supplement: Additional file 8: Figure S4. — Barplot of the ancestry coefficient q at K = 2, 3 and 4 of A) all nine loci and B) neutral loci. (PDF 751 kb) [file 12862_2016_599_MOESM8_ESM.pdf]
